# Neutrophil extracellular traps aggravate neuronal apoptosis and neuroinflammation via neddylation after traumatic brain injury

**DOI:** 10.7150/thno.111512

**Published:** 2025-06-20

**Authors:** Xu Zhang, Jianye Xu, Yonggang Fan, Guihong Shi, Bo Chen, Anni Wang, Yanlin Zhu, Lei Li, Haoran Jia, Dilmurat Gheyret, Jinchao Wang, Yiyao Cao, Shenghui Li, Xin Chen, Jianning Zhang, Shu Zhang

**Affiliations:** 1 School of Medicine, Nankai University, Tianjin, China.; 2 Tianjin Neurological Institute, Key Laboratory of Post Neuro-Injury Neuro-Repair and Regeneration in Central Nervous System, Ministry of Education and Tianjin City, Tianjin Medical University General Hospital, Tianjin, China.; 3 Department of Neurosurgery, Tianjin Medical University General Hospital, Tianjin, China.; 4 Department of Neurosurgery, The First Affiliated Hospital, College of Medicine, Zhejiang University, Hangzhou, Zhejiang, China.; 5 State Key Laboratory of Experimental Hematology, Tianjin, China.; 6 Beijing Neurosurgical Institute, Beijing Tiantan Hospital, Capital Medical University, Beijing, 100070, China.; 7 Institute of Brain and Brain-Inspired Science, Qilu Hospital, Shandong University and Shandong Key Laboratory of Brain Health and Function Remodeling, Jinan, China.

**Keywords:** NEDD8, Neddylation, MLN4924, Neutrophil extracellular traps, traumatic brain injury

## Abstract

**Rationale:** Neddylation, akin to ubiquitination, regulates various cellular processes by attaching neuronal precursor cell-expressed developmentally down-regulated protein 8 (NEDD8) to target proteins. Its specific role in traumatic brain injury (TBI) remains poorly defined. Neutrophil extracellular traps (NETs), which accumulate at injury sites in TBI models, are linked to poor outcomes, yet the connection between NETs and neddylation remains unclear.

**Methods:** We analyzed contused brain tissues from TBI patients and mice subjected to controlled cortical impact (CCI) using Western blotting, immunofluorescence, and immunohistochemistry. Neddylation was inhibited using MLN4924, a small-molecule NEDD8-activating enzyme (NAE) inhibitor. We conducted short-term neurobehavioral tests, Fluoro-Jade C staining, TUNEL assay, and Evans blue extravasation. Additionally, co-immunoprecipitation (Co-IP) and mass spectrometry were employed to explore the mechanisms of neddylation post-TBI.

**Results:** Neddylation increased in neurons during the acute phase of TBI. Inhibition of neddylation with MLN4924 reduced neuronal death, mitigated proinflammatory polarization of microglia, and maintained the integrity of the blood-brain barrier (BBB). MLN4924 treatment also decreased cerebral lesion volume and improved short-term neurological outcomes. NETs induced neuronal neddylation and apoptosis, while MLN4924 rescued neurons from NET-induced apoptosis. Mechanistically, NETs promoted NEDD8 binding to the ubiquitin ligase TRIM56, facilitating STING K63-linked ubiquitination and activating the NF-kB pathway, leading to neuroinflammation and neuronal death.

**Conclusions:** Our study revealed that NETs trigger neuronal death and neuroinflammation via neddylation. We propose that inhibiting neddylation could offer therapeutic benefits in TBI.

## Introduction

Traumatic brain injury (TBI) is a leading cause of neurological disorders and presents a significant public health challenge 1. Despite extensive research, clinical advancements have been limited due to the complex and heterogeneous nature of TBI 2, which causes tissue damage and neurological impairment through direct mechanical injury (primary injury) and subsequent cellular and biochemical changes (secondary injury). Secondary injury involves neuronal cell death and neuroinflammation, which interact and exacerbate each other 3. Current therapeutic options for post-traumatic neuronal death and persistent neuroinflammation are limited and largely ineffective, highlighting the need for a better understanding of the pathophysiology of secondary brain injury and the development of more targeted treatments.

The neddylation pathway, like ubiquitination, plays a critical role in various cellular processes. It attaches neuronal precursor cell-expressed developmentally down-regulated protein 8 (NEDD8) to target proteins 4. Like ubiquitination, neddylation is a three-step enzymatic process catalyzed by the NEDD8-activating enzyme E1 (NAE1 and UBA3), the NEDD8-conjugating enzyme E2 (UBC12), and several E3 enzymes 5, 6. Neddylation inhibition confers neuroprotection and blood-brain barrier (BBB) preservation in ischemic stroke and mitigates autophagic flux disruption, thereby exerting cardioprotective effects in myocardial ischemia-reperfusion injury. Additionally, it attenuates liver inflammation and suppresses Kupffer cell activation in hepatic fibrosis 7-9. Neddylation substrates include cullin family members and non-cullin proteins, such as p73 and TRIM21 10-12. However, whether neddylation occurs in TBI, what factors drive this modification, and which substrates are involved remains unclear.

Neutrophils, the most abundant circulating leukocytes and the first immune cells to reach the injured brain, play a key role in regulating neuroinflammation and neuronal apoptosis following brain injury 13, 14. Several mechanisms of neutrophil-mediated immune responses have been proposed, including neutrophil extracellular traps (NETs), web-like networks composed of DNA fibers and intracellular proteins, such as histones, myeloperoxidase (MPO), and other antimicrobial proteins. NETs are key contributors to inflammation, thrombosis, and autoimmune disorders 15-18. Peptidylarginine deiminase 4 (PAD4) is essential for chromatin decondensation through histone citrullination, facilitating NET extrusion 19. Our previous study demonstrated that NETs are markedly elevated following TBI, and that inhibition of NETs alleviates neuronal apoptosis 20, 21, suggesting a critical role for NETs in neuronal cell death after TBI.

Furthermore, neddylation has been implicated in the pathogenesis of various diseases 22. To the best of our knowledge, this study is the first to report an increase in neddylation levels following TBI. A previous study has reported that inhibition of neddylation could attenuate CCl4-induced cell apoptosis 9, indicating its involvement in apoptotic pathways. Given that both NETs and neddylation contribute to regulating apoptosis, we hypothesized that a regulatory relationship may exist between them. Furthermore, previous research has shown that NETs enhance macrophage pyroptosis by preventing the ubiquitination-mediated degradation of NLRP3 23. Considering that neddylation also plays a role in the ubiquitination process 12, we speculated that NETs may influence neddylation activity.

In this study, we explored the role of neddylation in TBI, and the regulatory influence of NETs on neddylation and its underlying mechanisms. Our findings identify potential therapeutic targets for the treatment of TBI.

## Results

### Neddylation levels are predominantly elevated in neurons of TBI patients and mice

**Figure 1:**
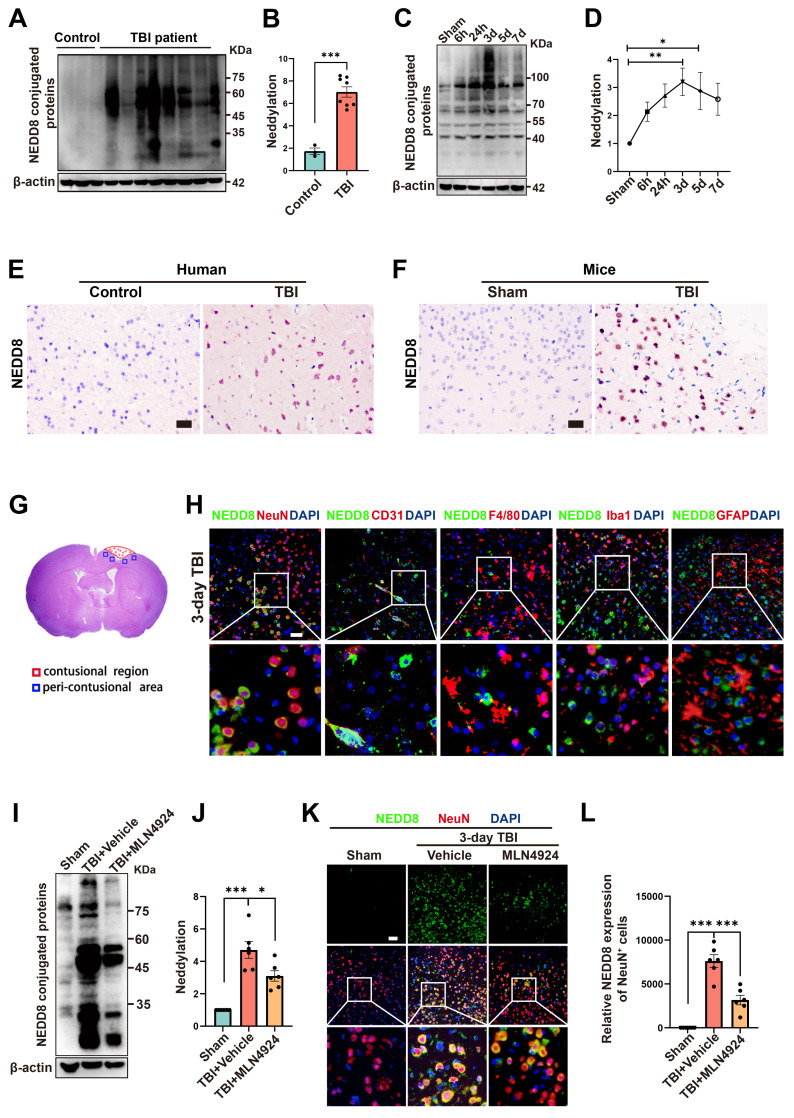
**Neddylation level is up-regulated after TBI.** (A-B) Immunoblot analysis and quantification of neddylation in brain tissues from controls (n = 3) and TBI patients (n = 8). Statistical analysis was performed using an unpaired Student's t-test. (C-D) Immunoblot analysis and quantification of neddylation in cortical tissue from sham-operated and CCI model mice at 6h, 24h, 3d, 5d, and 7d post-operation. n = 6 per group. Statistical comparisons among multiple groups were performed using one-way ANOVA test. (E) Representative IHC images of NEDD8 in human brain tissues. Scale bar = 50 μm. (F) Representative IHC images of NEDD8 in mice brain tissues. Scale bar = 50 μm. (G) Representative HE staining of coronal sections in mouse models of TBI. (H) Representative images of the colocalization of NEDD8 (green) with neurons (NeuN, red), endotheliocytes (CD31, red), macrophages (F4/80, red), microglia (Iba-1, red) and astrocytes (GFAP, red) at the lesion site at 3 d after TBI. Nuclei were stained with DAPI (blue). Scale bar = 50 μm. (I-J) Immunoblot analysis and quantification of neddylation in sham, TBI+ Vehicle, and TBI+MLN4924 groups. Statistical comparisons among multiple groups were performed using one-way ANOVA test. (K) Representative images of IF staining of NEDD8 (green) and NeuN (red). Nuclei were stained with DAPI (blue). Scale bar = 50 μm. (L) Quantitative analysis of NEDD8 expression of NeuN^+^ neurons. n = 6 per group. Statistical comparisons among multiple groups were performed using one-way ANOVA test. *P < 0.05, **P < 0.01, ***P < 0.001, Data are presented as mean values ± SEM.

To investigate neddylation following TBI, we collected epileptogenic tissues from 3 patients, brain tissues from 8 TBI patients (Table S5), and cortical tissue from CCI model mice at 6 and 24 h, 3, 5, and 7 days post-injury. We observed significantly elevated neddylation levels in TBI patients compared to controls (Figure 1A-B, unpaired Student's t-test). In TBI mice, neddylation levels peaked on day 3 (Figure 1C-D, one-way ANOVA test), with a marked increase in NEDD8-activating enzyme E1 subunits (NAE1 and UBA3) and the NEDD8-conjugating enzyme E2 (UBC12), although the increase in NAE1 was not statistically significant (Figure S1, one-way ANOVA test).

Immunohistochemistry revealed a significant increase in NEDD8-positive cells in TBI patients and mice compared to controls (Figure 1E-F). Immunofluorescence colocalization staining around the lesion site on day 3 post-TBI in mice showed that NEDD8 was primarily expressed in NeuN^+^ neurons, consistent with the findings in Alzheimer's patients 24. Moderate expression was also observed in CD31^+^ vascular endothelial cells, which is in line with findings from a stroke study 7. In contrast, little NEDD8 expression was detected in F4/80^+^ macrophages, Iba1^+^ microglia, or GFAP^+^ astrocytes (Figure 1G-H). Based on these results, we investigated neuronal neddylation and its underlying mechanisms. We inhibited the neddylation pathway using the specific NAE inhibitor MLN4924 25, the administration of which to TBI mice reduced neddylation levels in the peri-lesional brain tissue and neurons (Figure 1I-L, one-way ANOVA test).

### MLN4924 reduces lesion size and improves TBI-induced cognitive and motor deficits

**Figure 2:**
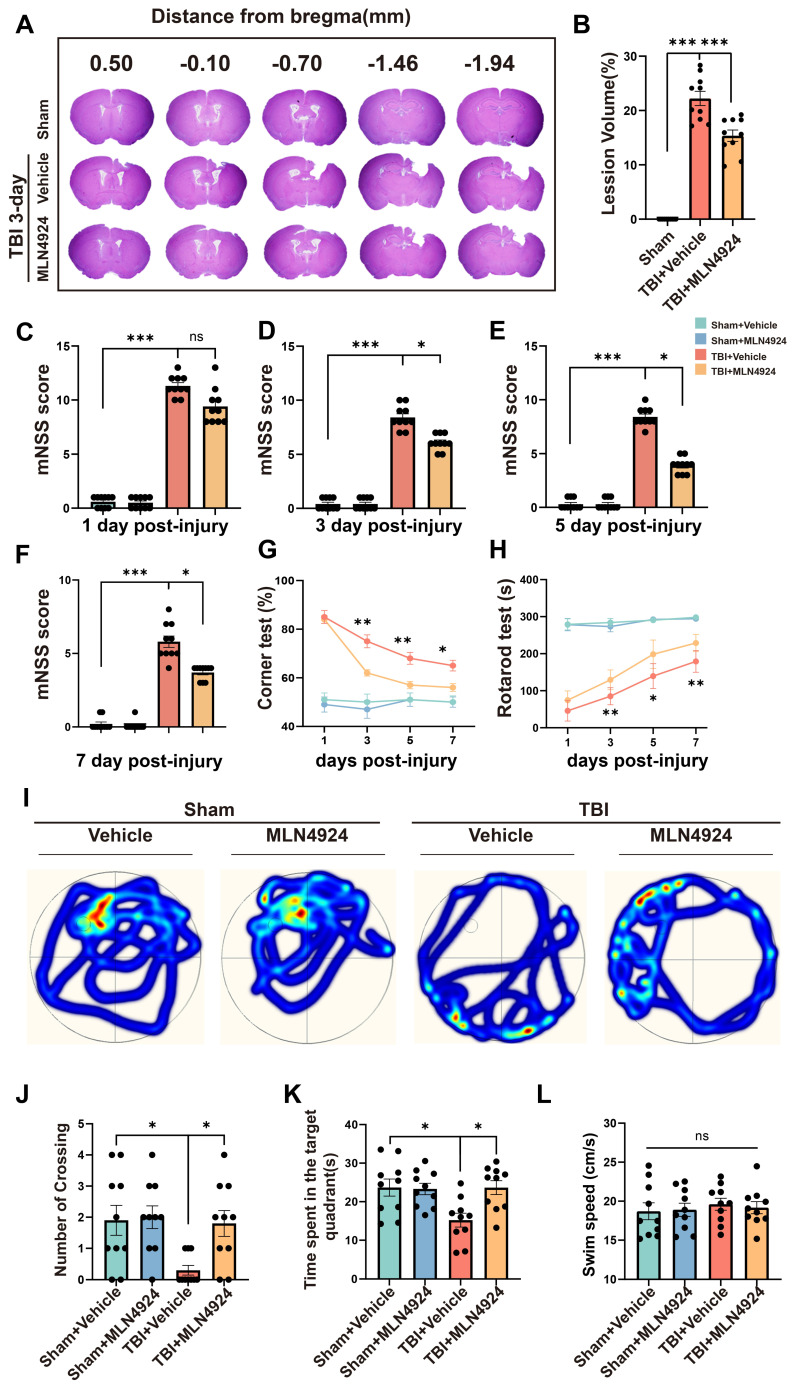
** Effects of MLN4924 treatment on cerebral lesion volume, cognitive and motor dysfunction caused by TBI.** (A) Representative images of serial coronal sections showing tissue loss at 3 days post-TBI. (B) Quantitative analysis of tissue loss. n = 10 per group. Statistical comparisons among multiple groups were performed using one-way ANOVA test. (C-F) mNSS score at 1, 3, 5, 7 days post injury. n = 10 per group. Kruskal-Wallis test was used to assess differences among groups at each time point. (G-H) Corner test (G) and rotarod test (H) at 1, 3, 5, 7 days post injury. n = 10 per group. Two-way repeated measures ANOVA was used to analyze differences across time points and treatment groups. (I) Representative heatmap of mice in the MWM test. (J-L) Number of crossing (J), time spent in the target quadrant (K) and swimming speed (L) in MWM test. n = 10 per group. Statistical comparisons among multiple groups were performed using one-way ANOVA test. *P < 0.05, **P < 0.01, ***P < 0.001. Data are presented as mean values ± SEM.

We assessed the impact of MLN4924 on acute lesion size post-TBI, by performing HE staining on serial coronal sections (Figure 2A). Results showed that MLN4924 reduced tissue loss at 3 days post-TBI (Figure 2B, one-way ANOVA test). The protective effects of MLN4924 on neurological function after TBI, were examined by conducting behavioral tests, including the modified neurological severity score (mNSS), rotarod, corner and Morris water maze (MWM) tests. The mNSS test showed gradual improvement in neurological functions following TBI. On days 3, 5, and 7 post-TBI, MLN4924-treated mice demonstrated significantly less motor dysfunction than the TBI + vehicle group, with a clear statistical difference (Figure 2C-F, Kruskal-Wallis test). In the corner test, the TBI + MLN4924 group showed significantly enhanced motor coordination compared to the TBI group on days 3, 5, and 7 (Figure 2G, two-way RM ANOVA test). The TBI + MLN4924 group demonstrated markedly enhanced performance in the rotarod test at 3, 5, and 7 days post-TBI compared to the TBI group (Figure 2H, two-way RM ANOVA test).

We used the MWM test to investigate learning and spatial memory function by conducting training sessions on days 15-19 post-TBI, followed by the MWM test on day 20 26. During the probe trial on day 20, the MLN4924-treated group crossed the previous platform location more frequently (Figure 2I-J, one-way ANOVA test) and spent more time in the target area (Figure 2K, one-way ANOVA test) after removing the platform. Swimming speed did not differ significantly between groups (Figure 2L, one-way ANOVA test). Thus, MLN4924 reduced lesion size and improved behavioral outcomes in TBI mice.

### MLN4924 inhibits neuronal apoptosis and neuroinflammation following TBI

**Figure 3:**
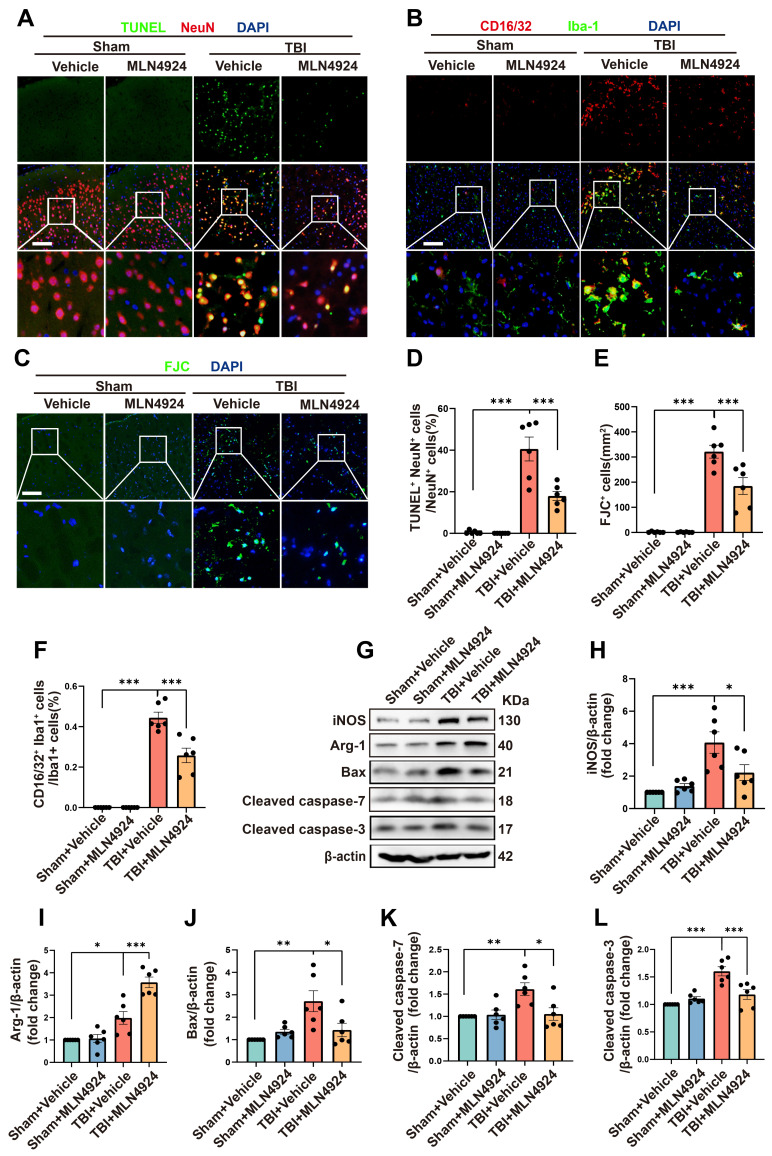
** Effects of MLN4924 treatment on neuronal apoptosis and neuroinflammation following TBI.** (A) Representative images of TUNEL (green) colocalization with neurons (NeuN, red). Nuclei were stained with DAPI (blue). Scale bar = 100 μm. (B) Representative images of Iba-1 (green) colocalization with CD16/32 (red). Nuclei were stained with DAPI (blue). Scale bar = 100 μm. (C) Representative images of the FJC (green) staining in the lesion at 3 days after TBI. Nuclei were stained with DAPI (blue). Scale bar = 100 μm. (D-F) Quantitative analysis of TUNEL-positive neurons (D), FJC -positive cells (E) and Iba1^+^ /CD16/32^+^ microglia (F). n = 6 per group. (G) Immunoblot analysis of iNOS, Arg-1, Bax, Cleaved caspase-7 and Cleaved caspase-3 expression. (H-L) Quantitative analysis of iNOS (H), Arg-1 (I), Bax (J), Cleaved caspase-7 (K) and Cleaved caspase-3 (L). n = 6 per group. *P < 0.05, **P < 0.01, ***P < 0.001. Statistical comparisons among multiple groups were performed using one-way ANOVA test. Data are presented as mean values ± SEM.

Inhibition of neddylation has been shown to mitigate damage from myocardial ischemia-reperfusion 8, ischemic stroke 7, and liver fibrosis, as well as to alleviate bile acid and CCl4-induced apoptosis 9. It also exerts anti-inflammatory effects in atherosclerosis by promoting macrophage polarization to the M2 phenotype 27. However, the role of MLN4924 in TBI remained unclear. Given that NEDD8 is primarily expressed in neurons, we first explored the effects of MLN4924 on neuronal apoptosis following TBI. TUNEL and FJC staining indicated that neddylation inhibition alleviated neuronal apoptosis and degeneration post-TBI (Figure 3A, C-E, one-way ANOVA test). Western blot analysis of peri-lesional brain tissue in TBI mice showed a significant increase in pro-apoptotic markers Bax, cleaved caspase-3, and 7 post-TBI. MLN4924 administration effectively reduced the expression of these pro-apoptotic factors (Figure 3G, J-L, one-way ANOVA test).

Next, we explored the effects of MLN4924 on neuroinflammation. Following brain injury, we observed a significant increase in CD16/32^+^, a classic proinflammatory marker, and Iba1^+^- positive microglia. MLN4924 significantly inhibited this increase (Figure 3B, F, one-way ANOVA test). Western blot analysis of the cortical tissue around the lesion revealed that MLN4924 inhibited the TBI-induced elevation of the pro-inflammatory factor iNOS and improved the reduction of the anti-inflammatory factor Arg-1 following TBI (Figure 3G-I, one-way ANOVA test). These results indicated that MLN4924 can improve neuronal apoptosis and neuroinflammation following TBI.

### Neddylation inhibition alleviates BBB leakage

**Figure 4:**
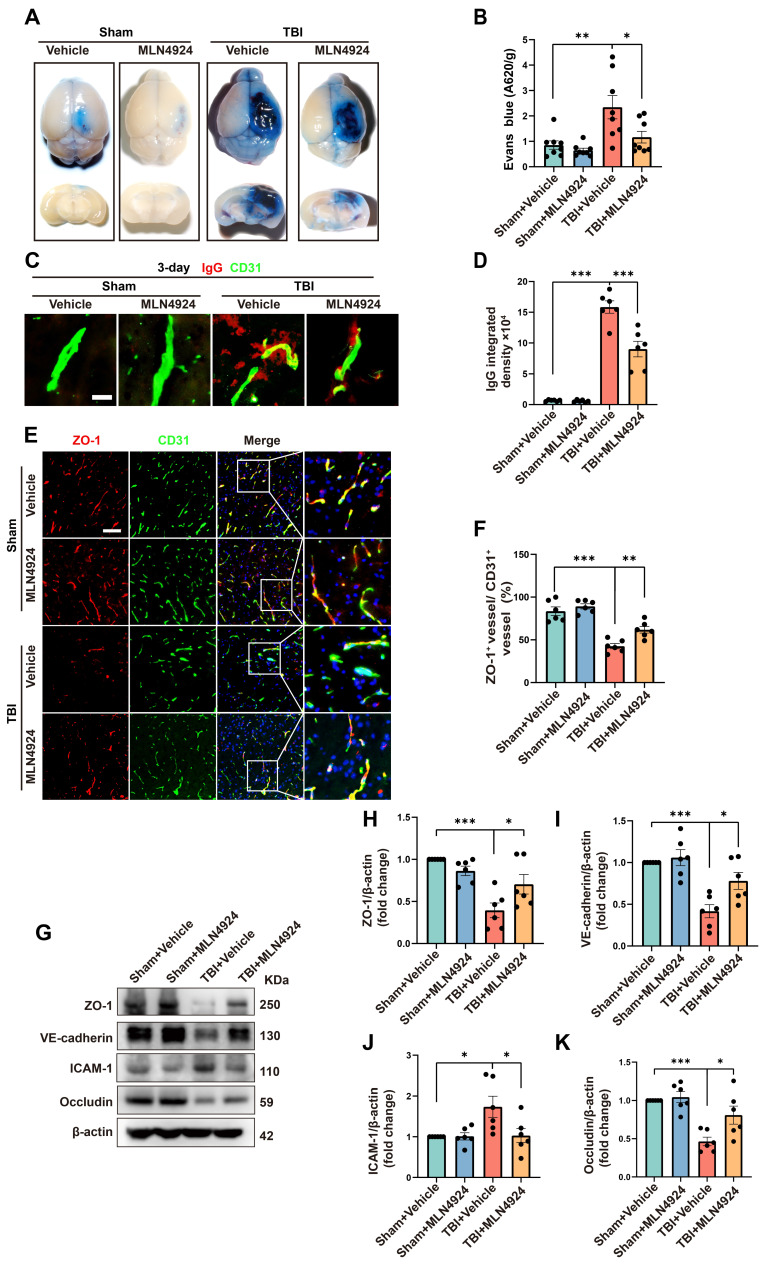
** Effects of MLN4924 treatment on BBB disruption post-TBI.** (A) Representative axial and coronal images of the brain following EB injection. (B) Quantitative analysis of EB leakage. n = 8 per group. (C) Representative images of extravascular IgG (red) deposits, with CD31 (green) staining indicating vessels. Scale bar = 10 μm. (D) Quantitative analysis of IgG integrated density. n = 6 per group. (E) Representative images of double immunofluorescence staining for ZO-1 (red) and vessels (CD31, green) 3 days post-TBI. Scale bar = 100 μm. (F) Quantitative analysis of ZO-1 expression of CD31^+^ vessels. n = 6 per group. (G)Immunoblot analysis of ZO-1, VE-cadherin, Occludin and ICAM-1 expression. (H-K) Quantification of the expression levels of ZO-1, VE-cadherin, Occludin and ICAM-1. n = 6 per group. *P < 0.05, **P < 0.01, ***P < 0.001. Statistical comparisons among multiple groups were performed using one-way ANOVA test. Data are presented as mean values ± SEM.

In addition to neuronal apoptosis and neuroinflammation, BBB leakage commonly occurs following brain injury, exacerbating secondary damage after TBI 28, 29. We investigated the role of MLN4924 in BBB leakage by injecting Evans Blue dye into sham and TBI mice on day 3 post-injury (Figure 4A). The TBI group showed a significant increase in dye leakage into the brain parenchyma, which was markedly reduced following MLN4924 treatment (Figure 4B, one-way ANOVA test). We next examined brain leakage of the endogenous blood-derived protein IgG. Immunostaining for IgG and the endothelial marker CD31 confirmed a significant increase in perivascular IgG deposits following TBI, which was markedly reduced in MLN4924-treated mice (Figure 4C-D, one-way ANOVA test). It has been reported that BBB permeability is regulated by endothelial junctions, which are diminished after TBI, resulting in barrier disruption 30.

We investigated whether MLN4924 treatment affects the expression of BBB junctional proteins following TBI. In the contused cortex, ZO-1^+^ endothelial junctions were significantly reduced 3 days post-TBI. However, MLN4924 treatment significantly increased ZO-1^+^ vessels compared to the TBI + Vehicle group (Figure 4E-F, one-way ANOVA test). Western blotting results showed that TBI significantly reduced the expression of the tight junction proteins ZO-1 and Occludin, as well as vascular endothelial cadherin (VE-cadherin), whereas MLN4924 treatment markedly restored these levels (Figure 4G-I, K, one-way ANOVA test). ICAM-1, a cell surface glycoprotein and adhesion receptor known for its role in leukocyte recruitment to inflammation sites 31, was also suppressed by MLN4924 following TBI (Figure 4G, J, one-way ANOVA test). In summary, our findings indicated that MLN4924 has protective effects on the blood-brain barrier and anti-inflammatory properties, consistent with results from a prior stroke study 7.

### NETs contribute to neddylation following TBI *in vivo* and *in vitro*

**Figure 5:**
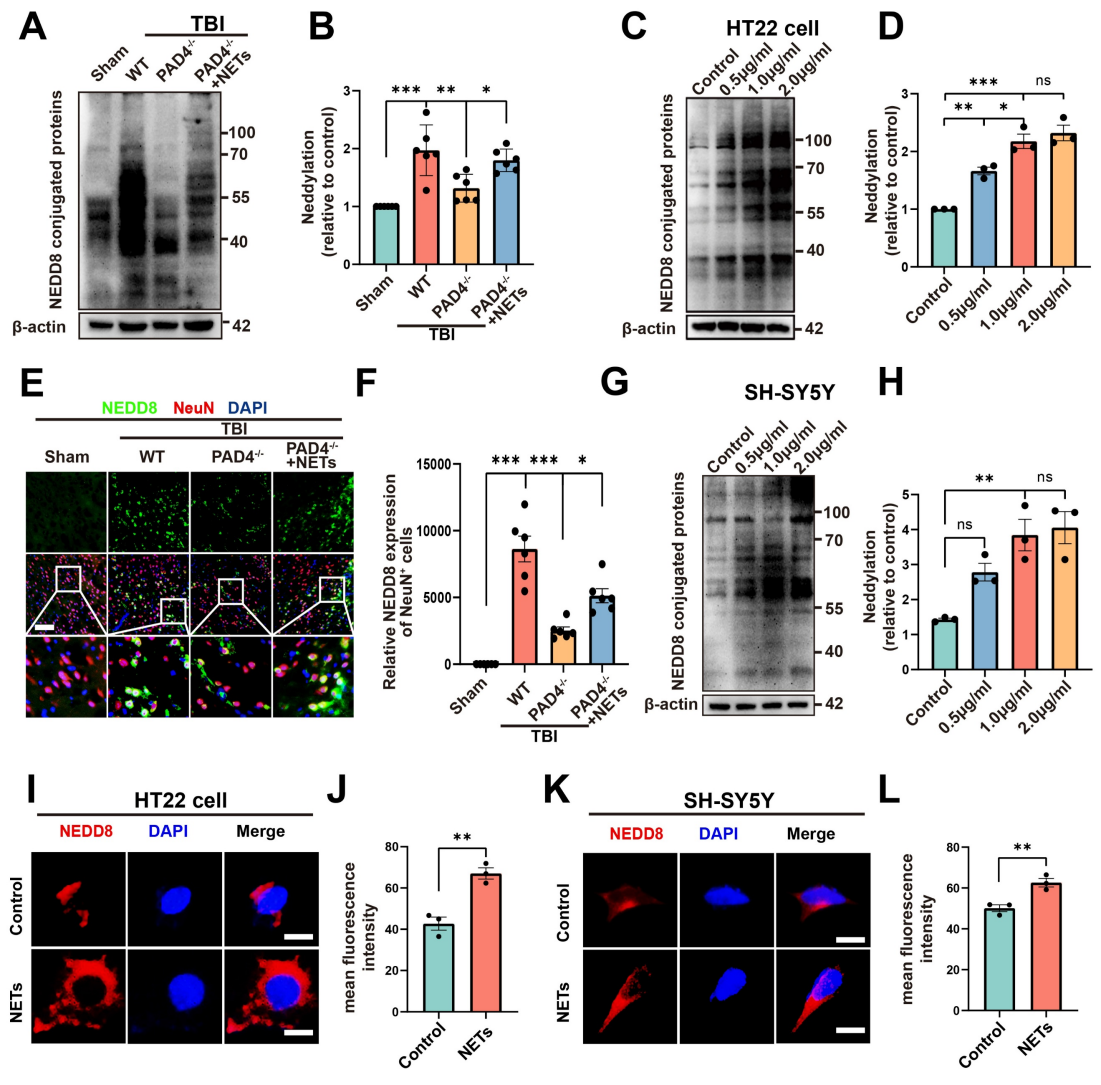
** Effects of NETs on neddylation level both *in vivo* and vitro.** (A) Immunoblot analysis of neddylation in sham, TBI+WT, TBI+PAD4^(-/-)^ and TBI+PAD4^(-/-)^+NETs groups. (B) Quantification of the expression levels of neddylation. n = 6 per group. Statistical comparisons among multiple groups were performed using one-way ANOVA test. (C) Immunoblot analysis of neddylation in HT22 cells treated with varying concentrations of mouse-derived NETs. (D) Quantification of the expression levels of neddylation in HT22 cells. n = 3 per group. Statistical comparisons among multiple groups were performed using one-way ANOVA test. (E) Representative images of IF staining of NEDD8 (green) and NeuN (red). Nuclei were stained with DAPI (blue). scale bar = 100 μm. (F) Quantitative analysis of NEDD8 expression of NeuN+ neurons. n = 6 per group. Statistical comparisons among multiple groups were performed using one-way ANOVA test. (G) Immunoblot analysis of neddylation in SH-SY5Y cells treated with different concentrations of human-derived NETs. (H) Quantification of the expression levels of neddylation in SH-SY5Y cells. n = 3 per group. Statistical comparisons among multiple groups were performed using one-way ANOVA test. (I) Representative images of NEDD8 expression in HT22 cells. scale bar = 20 μm. (J) Quantitative analysis of NEDD8 expression of HT22 cells. n = 3 per group. Statistical analysis was performed using an unpaired Student's t-test. (K) Fluorescent micrographs depicting NEDD8 protein expression in SH-SY5Y cells. scale bar = 20 μm. (L) Quantitative assessment of NEDD8 levels in SH-SY5Y cells. n = 3 per group. Statistical analysis was performed using an unpaired Student's t-test. *P < 0.05, **P < 0.01, ***P < 0.001, Data are presented as mean values ± SEM.

A substantial amount of NETs is formed following TBI 20, 32, as confirmed by our experimental results (Figure S2, unpaired Student's t-test). Peptidylarginine deiminase 4 (PAD4) is a key factor in NETs formation 33. To investigate whether NETs trigger neddylation, we established a PAD4^(-/-)^ mouse model to inhibit NET formation. Surprisingly, neddylation levels were significantly reduced in the TBI + PAD4^(-/-)^ group compared to the TBI group. However, after injecting NETs into the TBI + PAD4^(-/-)^ mice, the neddylation levels markedly increased (Figure 5A-B, one-way ANOVA test). Fluorescence colocalization analysis further demonstrated that neuronal neddylation levels were significantly lower in the TBI + PAD4^(-/-)^ group than in the TBI group. Notably, NETs injection effectively reversed this reduction, leading to a marked increase in neddylation levels (Figure 5E-F, one-way ANOVA test). These results suggested that NETs contributed to the increased neddylation levels following TBI.

We further validated this conclusion by conducting cellular experiments. HT22 cells were treated with 0.5, 1, or 2 µg/mL of NETs or with control media. Neddylation induced by mouse-derived NETs was assessed in HT22 cells using Western blotting. The results revealed significantly increased neddylation at 0.5, 1, and 2 µg/mL of NETs, with no significant difference between 1 and 2 µg/mL (Figure 5C-D, one-way ANOVA test). Neddylation in SH-SY5Y cells was significantly enhanced by human NETs at 1 and 2 µg/mL, with no significant variation between these concentrations (Figure 5G-H, one-way ANOVA test). Next, we added mouse-derived NETs to HT22 cell cultures at 1 µg/mL and for 24 hours. Fluorescence analysis showed that neddylation in NET-treated HT22 cells was significantly higher than in the control group (Figure 5I-J, unpaired Student's t-test). Next, validation in SH-SY5Y cells showed increased neddylation levels upon adding NETs (Figure 5K-L, unpaired Student's t-test).

### NETs promote neuronal apoptosis, neuroinflammation and BBB leakage through neddylation

**Figure 6:**
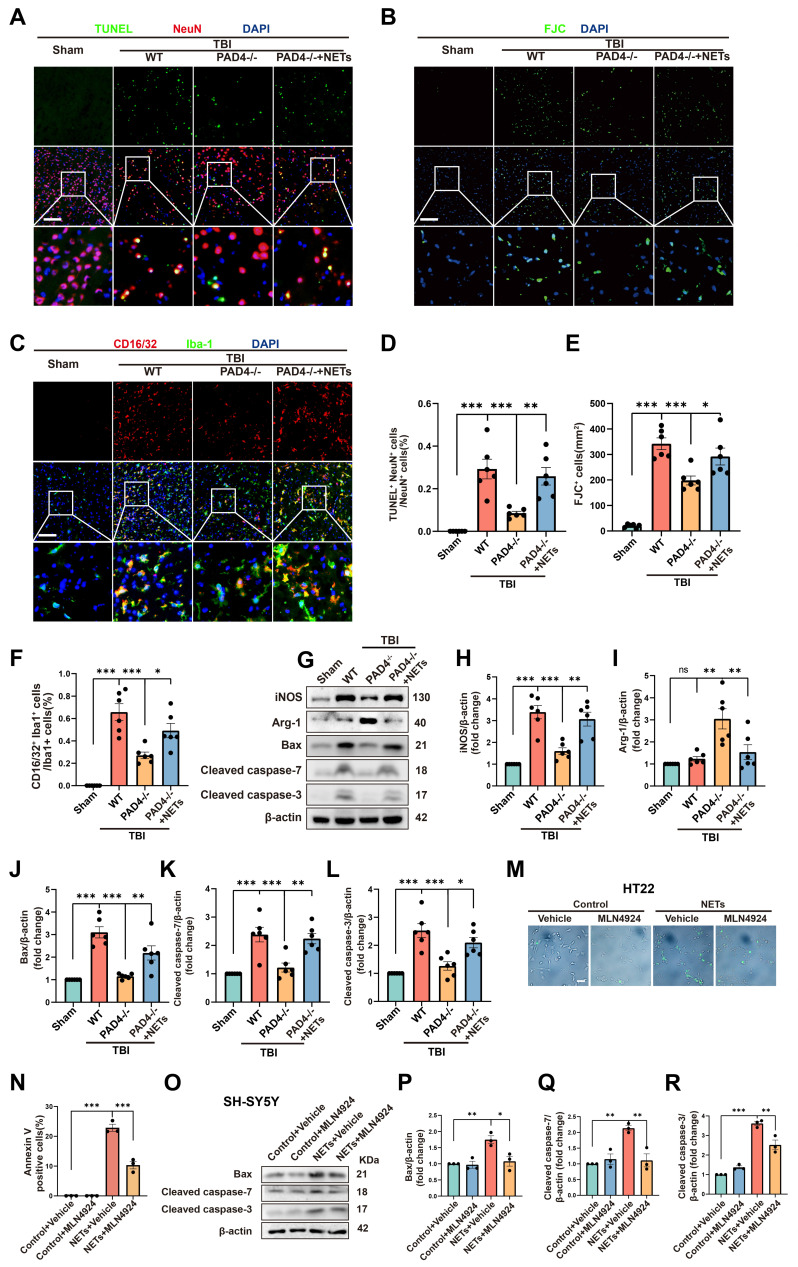
** NETs regulate neuronal apoptosis through neddylation.** (A) Representative images of TUNEL (green) colocalization with neurons (NeuN, red) in sham, TBI+WT, TBI+PAD4^(-/-)^ and TBI+PAD4^(-/-)^+NETs groups. Scale bar = 100 μm. (B) Representative images of FJC (green) staining in the lesion, with nuclei stained by DAPI (blue). Scale bar = 100 μm. (C) Representative images showing colocalization of Iba-1 (green) with CD16/32 (red), with nuclei stained by DAPI (blue). Scale bar = 100 μm. (D-F) Quantitative analysis of TUNEL-positive neurons (D), FJC -positive cells (E) and Iba1^+^ /CD16/32^+^ microglia (F). n = 6 per group. (G) Immunoblot analysis of iNOS, Arg-1, Bax, Cleaved caspase-7 and Cleaved caspase-3 expression in sham, TBI+WT, TBI+PAD4^(-/-)^ and TBI+PAD4^(-/-)^+NETs groups. (H-L) Quantitative analysis of iNOS (H), Arg-1 (I), Bax (J), Cleaved caspase-7 (K) and Cleaved caspase-3 (L). n = 6 per group. (M) Representative images of Annexin V (green) staining in HT22 cells treated with NETs or NETs combined with MLN4924. Scale bar = 50 μm. (N) Quantitative analysis of the proportion of Annexin V-positive cells across different groups. n = 3 per group. (O) Immunoblot analysis of Bax, cleaved caspase-7, and cleaved caspase-3 expression in SH-SY5Y cells treated with NETs or NETs plus MLN4924. (P-R) Quantitative analysis of Bax (P), Cleaved caspase-7 (Q) and Cleaved caspase-3 (R). n = 3 per group. *P < 0.05, **P < 0.01, ***P < 0.001. Statistical comparisons among multiple groups were performed using one-way ANOVA test. Data are presented as mean values ± SEM.

To further authenticate the role of NETs in neuronal apoptosis and neuroinflammation, we conducted fluorescence and Western blotting experiments on Sham, TBI+ Wild Type (WT), TBI+PAD4^(-/-)^, and TBI+PAD4^(-/-)^ +NETs mouse groups. TUNEL and FJC staining indicated that PAD4 knockout alleviated neuronal apoptosis. However, upon NETs injection into TBI + PAD4^(-/-)^ mice, neuronal apoptosis significantly increased (Figure 6A, B, D-E, one-way ANOVA test). Co-staining for microglia (Iba1) and CD16/32 revealed reduced neuroinflammation in the TBI+PAD4^(-/-)^ group. However, upon NETs injection into the TBI + PAD4^(-/-)^ mice, neuroinflammation was exacerbated again (Figure 6C, F, one-way ANOVA test). These findings are consistent with our previous study showing that Cl-amidine, a PAD4 inhibitor, mitigates neuronal apoptosis and neuroinflammation 20. Subsequently, we performed Western blotting and found that PAD4 knockout mice exhibited decreased levels of the proinflammatory marker (inducible nitric oxide synthase, iNOS) and increased anti-inflammatory marker (Arginase-1, Arg-1) compared to WT mice after TBI. In the TBI+PAD4^(-/-)^ group, the elevation of apoptosis markers, Cleaved-caspase7, Cleaved-caspase3, and Bax, was alleviated following TBI. However, these protective effects were reversed upon NETs injection into the TBI + PAD4^(-/-)^ mice, leading to increased neuroinflammation and apoptosis (Figure 6 G-L, one-way ANOVA test).

The present study demonstrated that NETs could increase neddylation levels and cause neuronal apoptosis. It was previously reported that inhibiting neddylation alleviated cell death 9. It was, therefore, necessary to investigate whether inhibiting neddylation could alleviate NET-induced neuronal death. We performed Annexin V-FITC and SYTOX Green fluorescence assays using HT22 cells.

We observed a significant increase in cell death at 1 µg/mL NETs concentration, indicated by the presence of Annexin V and SYTOX-positive cells. After treatment with MLN4924, the number of Annexin V and SYTOX-positive cells significantly decreased (Figure 6M-N and Figure S3A-B, one-way ANOVA test). In addition, siRNAs targeting NEDD8 were utilized to probe its role in NETs-induced apoptosis. HT22 cells were exposed to NETs (1µg/mL) and transfected with either si-NEDD8 or si-control or maintained in control media. SYTOX staining indicated that NEDD8 suppression mitigated NET-induced HT22 cell death (Figure S3C-D, one-way ANOVA test). To substantiate this conclusion, we conducted Western blotting using SH-SY5Y cells. We found that the NETs + MLN4924 group markedly diminished the apoptosis markers Cleaved-caspase-7, Cleaved-caspase-3, and Bax that were elevated by NETs (Figure 6O-R, one-way ANOVA test). Therefore, we concluded that inhibiting neddylation can alleviate NETs-induced apoptosis.

We investigated the impact of neuronal neddylation on the neuroinflammatory response by treating neurons with NETs, either alone or in combination with the neddylation inhibitor MLN4924, for 24 hours. Then, the medium was removed and replaced with a fresh culture medium. The conditioned medium was collected and applied to microglia for overnight incubation the following day. Subsequently, microglia were immunestained for CD16/32 and Iba-1. NETs-stimulated neuronal neddylation significantly increased the proportion of CD16/32⁺/Iba-1⁺ microglia, indicating enhanced inflammatory activation. This effect was attenuated by MLN4924 treatment, suggesting that neddylation contributes to microglia-mediated neuroinflammation (Figure S4, one-way ANOVA test).

Next, we investigated whether neuronal neddylation influences BBB integrity. NETs, alone or in combination with the neddylation inhibitor MLN4924, were added to neuronal cultures for 24 hours. Following treatment, the medium was replaced with fresh culture medium. After 24 hours, the conditioned medium was collected and applied to endothelial cells or astrocytes for overnight incubation. Endothelial cells were immune-stained for ZO-1 to assess tight junction integrity, while astrocytes were stained for MMP9 to evaluate BBB stability. NETs-induced neuronal neddylation led to a pronounced disruption of endothelial ZO-1 expression and a marked upregulation of MMP9 expression in astrocytes. Both effects were partially reversed by MLN4924 treatment (Figure S5, one-way ANOVA). Collectively, these findings demonstrated that neuronal neddylation exacerbates neuroinflammatory responses and compromises BBB integrity.

### NETs treatment enhances the interaction of NEDD8, TRIM56, and K63-linked ubiquitination of STING

**Figure 7:**
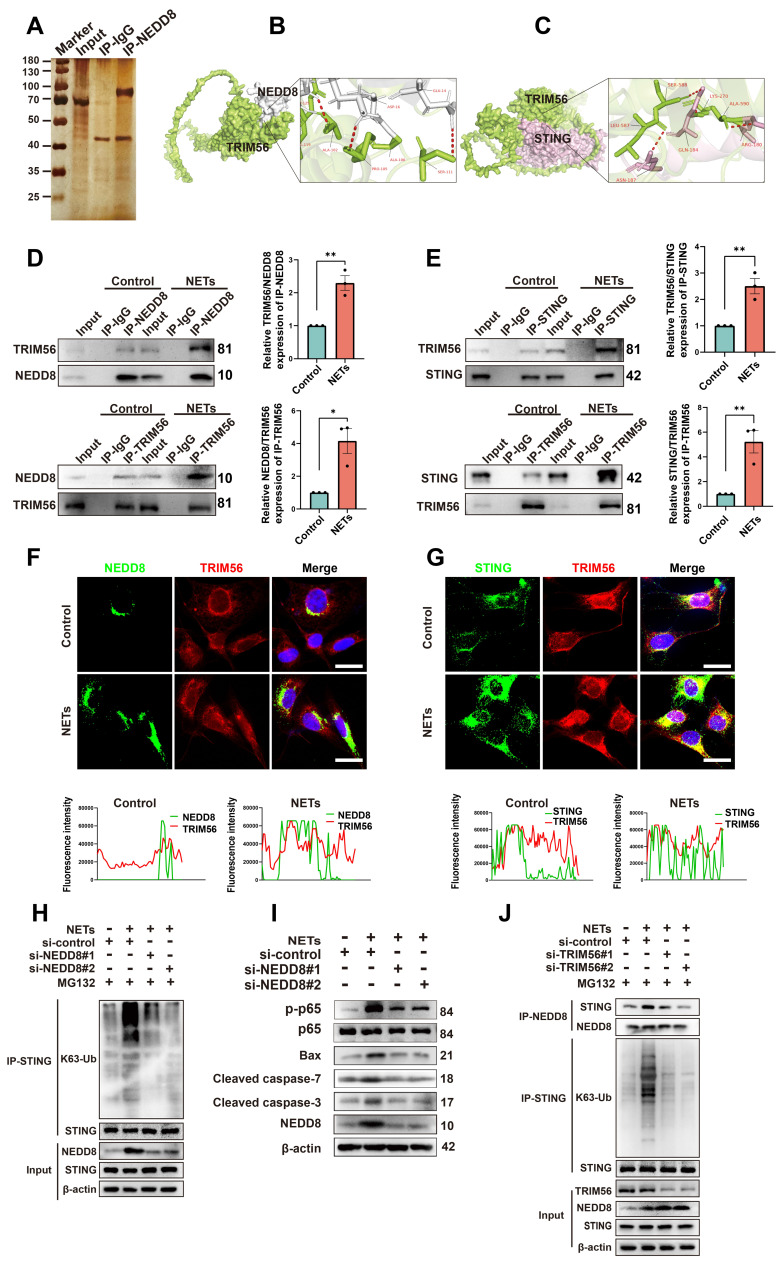
** NETs promote neddylation-induced K63-linked ubiquitination of STING via increased interaction with TRIM56.** (A) Silver staining of NEDD8-interacting proteins in NETs-treated HT22 cells, identified by mass spectrometry. (B) Human NEDD8-TRIM56 interaction binding model. NEDD8 is colored in gray, and TRIM56 is colored in green. (C) Binding model depicting the interaction between TRIM56 and STING of human. TRIM56 is colored in green, and STING is colored in light pink. (D) Co-IP analysis and quantification revealing the effect of NETs on the NEDD8-TRIM56 interaction in SH-SY5Y cells. n = 3 per group. (E) Co-IP analysis and quantification showing the effect of NETs on the TRIM56-STING interaction in SH-SY5Y cells. n = 3 per group. (F) Colocalization images of NEDD8 (green) and TRIM56 (red) with corresponding fluorescence intensity plots. Scale bar = 20 μm. (G) Colocalization images of STING (green) and TRIM56 (red) with corresponding fluorescence intensity plots. Scale bar = 20 μm. (H) Effects of NEDD8 on the K63-linked ubiquitination level of STING in SH-SY5Y cells, detected by Co-IP. MG132 (10 μM) pretreated cells for 6 hours. (I) Immunoblot analysis of p-p65, p65, Bax, Cleaved caspase-7, Cleaved caspase-3 and NEDD8 expression. (J) Effects of TRIM56 on the interaction between NEDD8 and STING and the K63-linked ubiquitination level of STING in SH-SY5Y cells, detected by Co-IP. MG132 (10 μM) pretreated cells for 6 hours. *P < 0.05, **P < 0.01, ***P < 0.001. Statistical analysis was performed using an unpaired Student's t-test. Data are presented as mean values ± SEM.

The specific mechanisms by which NETs induce neuronal apoptosis through neddylation were explored by Co-IP; we also used mass spectrometry to identify NEDD8-binding partners (Figure 7A). TRIM56, a protein involved in cell apoptosis 34, was highly enriched in NEDD8 antibody-bound samples. Docking results (https://gramm.compbio.ku.edu/) suggested a high likelihood of interaction between NEDD8 and TRIM56 (Figure 7B). Co-IP assays showed increased interaction between NEDD8 and TRIM56 in NET-treated cells (Figure 7D and Figure S6A, unpaired Student's t-test). It has been reported that TRIM56 interacts with STING and targets it for K63-linked ubiquitination upon stimulation 35. Our docking results further indicate a likely interaction between TRIM56 and STING (Figure 7C), with Co-IP assays showing an enhanced interaction between TRIM56 and STING in NET-treated cells (Figure 7E and Figure S6B, unpaired Student's t-test). Co-immunofluorescence and colocalization analysis revealed enhanced interactions between NEDD8 and TRIM56, as well as TRIM56 and STING, following NETs treatment, evidenced by increased overlap and channel coherence in the image analysis plots (Figure 7F-G). STING K63-linked ubiquitination triggered NF-kB pathway activation 36, which could induce neuroinflammation and cell apoptosis 37.

We found that NETs elevated STING K63-linked ubiquitination and activated the downstream NF-kB pathway, resulting in neuronal apoptosis. Knockdown of NEDD8 mitigated STING K63-linked ubiquitination and the level of neuronal apoptosis (Figure 7H-I and Figure S7, one-way ANOVA test). Additionally, NETs boosted NEDD8-STING binding, and TRIM56 knockdown decreased NEDD8-STING interaction and STING K63-linked ubiquitination (Figure 7J and Figure S8, one-way ANOVA test). To validate the NETs-NEDD8-STING axis *in vivo*, we performed immunofluorescence staining on the injury sites of TBI and TBI+PAD4^(-/-)^ mice. Notably, the number of NEDD8/STING double-positive neurons was significantly reduced in TBI+PAD4^(-/-)^ mice compared to TBI mice (Figure S9). This suggested that NETs promote neuronal NEDD8/STING activation *in vivo*, which may contribute to regulating neuronal apoptosis. In summary, our results showed that NETs enhance the NEDD8-TRIM56 interaction, promoting TRIM56-mediated STING K63 ubiquitination and subsequent STING-induced apoptosis.

### Zinc Finger of TRIM56 interacts with NEDD8 and STING

**Figure 8:**
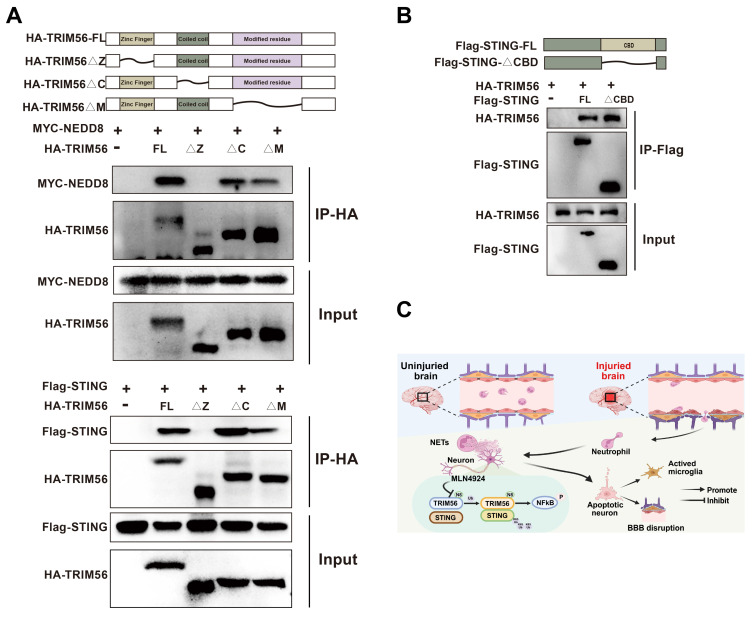
**Molecular interactions among NEDD8, TRIM56, and STING.** (A) Co-IP assays using anti-HA were conducted in 293T lysates transfected with MYC-NEDD8 or Flag-STING and indicated HA-TRIM56 constructs. (B) Co-IP assays using anti-Flag were performed on 293T lysates transfected with HA-TRIM56 and indicated Flag-STING constructs. (C) Graphical model of the study created using BioRender.

Protein domains, defined by unique spatial structures and independent functions, are essential in protein-driven biological processes. Their identification and functional analysis provide critical insights into protein rol**e**s 38. We generated a series of deletion mutants and performed co-immunoprecipitation assays to explore the potential interaction domains of NEDD8, TRIM56, and STING. Our results showed that the deletion of TRIM56's Zinc Finger domain disrupted its interactions with NEDD8 and STING (Figure 8A). Removal of the CBD domain in STING did not affect the interaction with TRIM56 (Figure 8B). Altogether, the Zinc Finger domain in TRIM56 is essential for binding to both NEDD8 and STING, providing valuable insights for developing therapeutic strategies to modulate these interactions. Figure 8C shows the graphical model of this study.

## Discussion

This study discovered elevated neddylation levels following TBI, with NETs contributing to this increase. Inhibition of neddylation levels mitigated neuroinflammation, neuronal apoptosis, blood-brain barrier leakage, and improved behavioral outcomes in TBI mice. Mechanistically, we found that NETs enhanced the binding between NEDD8 and the E3 ubiquitin ligase TRIM56, leading to activated TRIM56 promoting STING K63-linked ubiquitination and subsequent activation of the NF-kB pathway, thereby exacerbating neuroinflammation and neuronal apoptosis.

The neddylation process conjugates NEDD8 to target proteins, enabling specific biological functions. Treatment with MLN4924, an inhibitor of neddylation that targets E1 activity 39, suppresses NF-kB activation in macrophages, endothelial cells and B-cells 40-42. Furthermore, neddylation inhibition promotes the polarization of macrophages towards the M2 phenotype 27. Consistent with previous findings, we observed that inhibiting neddylation after TBI attenuated neuroinflammation and decreased the proportion of CD16/32^+^ microglia. Knockdown of NEDD8 inhibits NET-induced activation of the NF-kB pathway and subsequent neuronal apoptosis.

Following TBI, NETs are abundantly formed at the lesion site 32. Our study detected elevated neddylation levels after TBI, with neddylation predominantly occurring in neurons. It has been reported that NETs can induce NLRP3-dependent macrophage pyroptosis by deubiquitinating NLRP3, thereby reducing its degradation 23. This indicates that NETs can influence ubiquitination modifications. NEDD8, central to neddylation, typically binds to E3 ubiquitin ligases 43, but the effect of NETs on neddylation, which precedes ubiquitination, is yet to be determined. Unexpectedly, we found markedly lower neddylation levels post-TBI in PAD4^(-/-)^ mice than in their wild-type counterparts. Additionally, in HT22 and SH-SY5Y cells, neuronal neddylation increased with NETs concentration in a dose-dependent manner. Thus, our study provides the first evidence that NETs can elevate neuronal neddylation levels. Our prior work showed that NETs triggered neuroinflammation and neuronal apoptosis 20. Given that neddylation inhibition can mitigate apoptosis 9, we have demonstrated that NETs elevate neuronal neddylation. We hypothesized and subsequently confirmed that neddylation inhibition could ameliorate NET-induced neuronal apoptosis.

We found enhanced NEDD8-TRIM56 binding in NET-exposed cells via Co-IP and mass spectrometry and confirmed by co-immunofluorescence which elucidated how NETs triggered neuroinflammation and neuronal apoptosis via neddylation. TRIM56, a TRIM family E3 ligase, is crucial for inflammation, innate antiviral responses, and cancer development 44. TRIM56 binds STING upon pathogen challenge, promoting its K63 ubiquitination and activating the NF-kB pathway, which induces inflammation and cell death 35-37, 45, 46. In our study, molecular docking predicted the TRIM56-STING interaction, which was increased in NET-stimulated cells using Co-IP and co-immunofluorescence. NETs also heightened STING K63-linked ubiquitination, which was reduced by knocking down NEDD8 and TRIM56, inhibiting the NF-kB pathway and mitigating neuronal apoptosis.

Neddylation exerts a dual role in neurodegenerative diseases. On the one hand, it regulates Tau degradation, as neddylation inhibition accelerates cellular aging and promotes Tau aggregation and phosphorylation in neurons. This inhibition also leads to selective neuronal loss in both Alzheimer's (AD) and Parkinson's (PD) disease models 47. On the other hand, PARKIN and PINK1, key regulators of mitochondrial quality control, are neddylation substrates. Neddylation enhances the E3 ligase activity of PARKIN and stabilizes the 55 kDa proteolytic fragment, an active form of PINK1 48. Dysregulated neddylation of these proteins is thus implicated in PD pathogenesis 22, 49. We also found that neddylation promoted neuronal apoptosis in the acute phase of TBI. However, whether neddylation contributes to neuronal repair during the recovery phase remains to be further investigated.

The TRIM protein family possesses a conserved RBCC motif, comprising a RING zinc finger, one or two B-boxes, and a coiled-coil domain, followed by a variable C-terminal region. Most RING-finger-containing TRIM proteins serve as E3 ubiquitin ligases 50, 51. Our Co-IP results showed that the Zinc Finger (containing the RING-finger domain) of TRIM56 was necessary for its interaction with NEDD8 and STING, indicating that the Zinc Finger is an essential functional domain of TRIM56 as an E3 ubiquitin ligase. The CDN-binding domain (CBD) is a domain of STING 52, 53. Deleting CBD did not affect the binding between STING and TRIM56, as shown by Co-IP results. Further investigation is warranted to identify the functional domain mediating the STING-TRIM56 complex.

Our study has several limitations and potential clinical constraints. First, the role of neddylation in different age groups or female TBI mouse models needs to be assessed, which may influence the generalizability of our findings. Second, while we identified NETs as a contributor to increased neddylation levels following TBI, other regulatory factors remain to be explored. Additionally, the use of SH-SY5Y and HT22 cells to model neuronal pathology after brain injury cannot fully recapitulate the complexity of the *in vivo* environment. Several challenges must be addressed for the clinical application of MLN4924 in TBI. Given its broad impact on the neddylation system, potential off-target effects and toxicity require careful evaluation. Moreover, optimizing dosage and ensuring long-term safety necessitate extensive preclinical and clinical investigations to balance efficacy and adverse effects. Also, translational challenges, including interspecies differences and the variability of TBI pathology among patients, may impact its therapeutic applicability. One key challenge is its limited ability to penetrate the blood-brain barrier (BBB), which could hinder its effectiveness in targeting CNS pathology. To overcome this, nanocarrier-based delivery systems may be explored to enhance treatment specificity, improve drug delivery efficiency, and optimize therapeutic outcomes.

Our study demonstrated that NETs lead to increased levels of neddylation in neurons. NEDD8 promoted STING K63-linked ubiquitination by binding to the E3 ubiquitin ligase TRIM56, activating the NF-kB pathway, resulting in neuroinflammation and neuronal apoptosis following TBI. These findings suggest that neddylation inhibition and NETs suppression may offer promising therapeutic strategies for secondary brain injury in TBI patients.

## Materials and Methods

### Human samples

This study was conducted in accordance with the principles of the Declaration of Helsinki and was approved by the Ethics Committee of Tianjin Medical University (IRB2024-YX-536-01). Human clinical specimens were collected following an ethically reviewed protocol from Tianjin Medical University General Hospital. Tissues were obtained from eight patients with moderate to severe TBI and three controls from the Department of Neurosurgery, Tianjin Medical University General Hospital.

### Mice

All animal experiments were approved by the Institutional Animal Care and Use Committee of Tianjin Medical University General Hospital (No: IRB2020-DW-19). Pad4-/- mice were sourced from The Jackson Laboratory (Bar Harbor, USA) and were backcrossed for eight generations onto a C57BL/6J background at Tianjin Neurological Institute. The experiments utilized male mice aged 8 weeks. C57BL/6J mice (8 weeks, 22-24 g) were purchased from VitalRiver Laboratory Animal Technology Co., Ltd. (Beijing, China). Mice were acclimatized in a temperature- and humidity-controlled room (20-22°C, 50-55% humidity) under a 12-hour light/dark cycle, with ad libitum access to food and water. All animals were randomly assigned to experimental groups by a blinded coordinator.

### Experimental design

All outcomes were assessed by independent, blinded investigators. The experimental flow chart and experimental design are presented in Supplementary File Part 1.

### TBI model

TBI was induced using a digital electromagnetic CCI device (eCCI6.3, Customs Design & Fabrication, Richmond, USA), as previously described 54. In brief, mice were anesthetized with 1.5% isoflurane and positioned in a stereotaxic frame. A craniotomy was performed to create a 3.5 mm diameter skull window on the right parietotemporal region (2.5 mm lateral to the sagittal suture and 2.5 mm posterior to bregma), preserving the dura mater. A 3 mm flat-tip impactor was applied directly to the dura, with impact parameters set to a 2.2 mm depth, 200 ms dwell time, and 5 m/s velocity. Following the injury, mice were allowed to recover on a 37°C heat pad until fully awake. Mice in the sham group underwent the same craniotomy procedure without the CCI impact. To investigate the effect of MLN4924 on neddylation inhibition, mice were injected subcutaneously (s.c.) with 60 mg/kg MLN4924 or control solvent once daily, 1 hour after TBI, for three consecutive days. To examine the effect of NETs on neddylation, PAD4^⁻/⁻^ mice received daily tail vein injections of 60 µg/kg NETs for three consecutive days, starting 1 hour after TBI.

### Rotarod test

As previously described 55, limb motor coordination and balance in mice were evaluated using an accelerating Rota-rod apparatus (RWD Life Science, Shenzhen, China). Prior to CCI induction, mice underwent acclimation and training (first acclimation session: 0 rpm for 30 s, followed by three training sessions) across three consecutive days with three trials per day. Mice were initially trained at a slow rotational speed (4 rpm/min) for 1 minute, followed by an accelerating speed (from 4 to 40 rpm over 5 minutes), with a 30-minute rest between trials. On day 1 and day 3 post-TBI, each mouse was placed on the accelerating Rota-rod, which increased speed from 4 to 40 rpm/min over 5 minutes. The latency to fall was recorded for each mouse. Each mouse underwent three tests per day with the same speed, with a 30-minute interval between trials, and the average latency to fall was calculated for analysis. Two investigators, blinded to the group assignments, performed the tests.

### Neurological score assessment

Neurological function was assessed using the modified Neurological Severity Score (mNSS) 56. The mNSS includes tests for motor function (movement abnormalities and muscle state), sensory function (tactile and visual), balance, and reflexes. See Table S1 for details.

### Corner test

The corner test was utilized to assess sensorimotor and postural asymmetries in mice 57. Each mouse was positioned to face a 30° angle plastic board and the direction of their turn at the corner was documented. This procedure was repeated 10 times per mouse. Results were presented as the percentage of right turns, calculated as (right turn frequency/total trials) × 100%.

### Morris water maze

Mice were evaluated for spatial memory and learning using a Morris water maze in a 1.5 m diameter cylindrical tank filled with opaque water, divided into four quadrants. Mice were randomly initiated from any quadrant. If the submerged platform was not located within 90 seconds, the mice were guided to it and remained for 15 seconds. The latency to find the platform was documented. After 5 days of training, on day 6, the platform was removed. Mice were then allowed to swim freely, and the time to search for the platform's previous location was recorded and analyzed 26.

### Evans blue extravasation assay

Evans blue (2%, 4 mL/kg, Sigma Aldrich) in sterile saline was injected intravenously and allowed to circulate in mice for 2 hours before sacrifice. The mice then underwent transcardial perfusion with PBS, and their brains were dissected and weighed. The samples were homogenized in N,N-dimethylformamide (Sigma Aldrich) and incubated at 60 °C for 72 hours. After centrifugation, the absorbance of the supernatant was measured by spectrophotometry (Molecular Devices, Sunnyvale, CA) at an optical density of 620 nm.

### Fluoro‑Jade C staining

Nerve cell degeneration was detected using the FJC Ready-to-Dilute Staining Kit (FJC, TR-100-FJ, Biosensis, CA). Briefly, brain sections were sequentially immersed in 1% NaOH, 70% ethanol, distilled water, and 0.06% potassium permanganate. After rinsing with distilled water, sections were stained with 0.0001% FJC working solution and DAPI, then rinsed, dried in the dark, cleared in xylene, and coverslipped. Stained sections were observed, and images were captured using a fluorescence microscope (Olympus, Heidelberg, Germany).

### TUNEL staining

Apoptotic neurons were observed by double-staining with the neuronal marker NeuN (red) and TUNEL (green) using the In Situ Cell Death Detection Kit (Roche, South San Francisco, CA, USA) per the manufacturer's instructions. Nuclei were counterstained with DAPI (Abcam) and imaged with an inverted fluorescence microscope (Olympus, Japan).

### Neutrophil isolation and NETs incubation

Human neutrophils were extracted from whole blood using the MACSxpress® Whole Blood Neutrophil Isolation Kit (Miltenyi Biotec, 130-104-434) according to the protocol provided by the manufacturer. Mouse bone marrow neutrophils were isolated using the Neutrophil Isolation Kit (Miltenyi Biotec, 130-097-658) following the manufacturer's protocol. Isolated neutrophils were resuspended in RPMI-1640 medium (Thermo Fisher, 61870036), plated at 5 × 10⁵ cells per well in 12-well plates, and stimulated with 10 µg/mL LPS (Sigma-Aldrich, L2630) at 37°C for 2.5 hours 58. NETs were collected from the supernatant following vigorous mixing and centrifugation at 300 g for 5 minutes. NETs were quantified using the Picogreen dsDNA Assay Kit (Invitrogen) and EpiQuik Circulating Histone H3 Citrullination ELISA Kit.

### Cell culture

SH-SY5Y neuroblastoma cells (American Type Culture Collection, Rockville, MD, USA) and HT22 cells (Shanghai Institute of Cell Biology, China) were cultured in Dulbecco's Modified Eagle's Medium (DMEM; 11995065, Thermo Fisher, Waltham, USA), supplemented with 10% Fetal Bovine Serum (FBS; A5669401, Thermo Fisher) and 1% penicillin-streptomycin (Gibco, USA) in a 37°C incubator with 5% CO2. 293T cells (CRL-3216) were obtained from ATCC and cultured in Dulbecco's Modified Eagle's Medium (DMEM; 11995065, Thermo Fisher, Waltham, USA) supplemented with 10% Fetal Bovine Serum (FBS; A5669401, Thermo Fisher). MLN4924 (1 μM) or vehicle was added 30 minutes before addition of NETs.

### Annexin V fluorescence assay

Neutrophils were cultured in 6-well plates for 1 hour to evaluate extracellular DNA. HT22 cells were plated in 12-well plates and stimulated for 24 hours to analyze cell death. The medium containing NETs was then removed, and the cells were washed three times with PBS. Annexin V-FITC Apoptosis Detection Kit (C1062S, Beyotime) was applied. Images were captured with an Olympus fluorescence microscope, with Annexin V positivity marking apoptotic cells.

### SYTOX fluorescence assay

Neutrophils were cultured in 6-well plates for 1 hour to evaluate extracellular DNA. HT22 cells were plated in 12-well plates and stimulated for 24 hours to analyze cell death. The medium containing NETs was then removed, and the cells were washed three times with PBS. Following complete removal of NETs, 167 nM SYTOX Green Nucleic Acid Stain (S7020, Thermo Fisher) was applied. Images were captured with an Olympus fluorescence microscope, with SYTOX Green positivity marking dead cells.

### IHC staining

Formalin-fixed, paraffin-embedded brain sections from controls and TBI patients, as well as controls and TBI mice, were prepared. Sections were heated, deparaffinized, rehydrated, and treated with sodium citrate buffer (pH 6.0) for antigen retrieval. Endogenous HRP activity was blocked using 3% hydrogen peroxide (H₂O₂). Slides were blocked with 10% normal goat serum and incubated overnight at 4°C with primary antibodies (NEDD8, 2745S; CST). Signals were visualized using HRP-conjugated secondary antibodies with DAB as the substrate, followed by hematoxylin counterstaining. Representative images were captured using a fluorescence microscope.

### Immunofluorescence staining

Human brain tissues and mouse brains were fixed in 4% paraformaldehyde. The paraffin-embedded tissues were sectioned at 6 μm thickness, mounted on slides, and then underwent deparaffinization and antigen retrieval. For cell staining, SH-SY5Y and HT22 cells were fixed in 4% paraformaldehyde for 25 minutes. After washing, permeabilization, and blocking, cells were incubated overnight at 4°C with primary antibodies. This was followed by incubation with secondary antibodies and 4',6-diamidino-2-phenylindole (DAPI; ab104139, Abcam, Cambridge, UK) for 1 hour and 15 minutes, as previously described 59. For endogenous IgG leakage measurements, tissue sections were incubated overnight at 4°C with a combination of AlexaFluor594 donkey anti-mouse IgG and goat anti-CD31, followed by incubation with AlexaFluor488 donkey anti-goat IgG, as previously described 60. Staining was examined using a fluorescence microscope (IX73; Olympus, Tokyo, Japan), and images were analyzed with ImageJ (Version 1.46r, Wayne Rasband, USA). A list of all antibodies used in this assay is provided in Table S2.

### Western blot analysis

Protein samples from lysed tissues or cells were separated and transferred onto a polyvinylidene fluoride (PVDF) membrane (IPVH00010 or ISEQ00010, Millipore, Billerica, US) according to established protocols 61. After blocking to prevent nonspecific binding, the membrane was incubated with primary antibodies overnight at 4°C, followed by a 1-hour incubation with secondary antibodies. Protein bands were detected using a G: Box Chemi XT4 imaging system (Bio-Rad, Hercules, US) and quantified with ImageJ software. The list of antibodies used in this assay is provided in Table S3.

### Transfection with siRNAs or plasmids

The siRNAs were synthesized by RiboBio Co., Ltd. (Guangzhou, China), and plasmids were purchased from GenScript Biotech Corporation (Nanjing, China). Transfections of siRNAs or plasmids were performed using K2® transfection system (T060-1.0, Biontex) or Lipofectamine3000 (L3000015, Thermo Fisher) according to the manufacturer's instructions.

### Co‑IP assay

Co-IP assay was conducted using the Pierce Crosslink Magnetic Co-IP Kit (88805, Thermo Fisher) per the manufacturer's instructions. Cell lysates were incubated with antibody-bound beads overnight at 4°C. After washing and denaturation, target proteins were collected for analysis. Proteasomal activity was inhibited with MG132 (M8699, Sigma-Aldrich). All antibodies used in the co-IP and immunoblotting assays are listed in Table S4.

### Statistical analysis

All data were analyzed and reported as means ± standard error of the mean (SEM) using GraphPad Prism 9 (GraphPad Software, San Diego, CA, USA). For multiple comparisons, we applied one-way ANOVA with Tukey's test for equal SDs or the Brown-Forsythe and Welch ANOVA with Dunnett's T3 test for unequal SDs. Kruskal-Wallis test was used to assess mNSS data at each time point. Two-way Repeated Measures ANOVA (RM ANOVA) with Geisser-Greenhouse correction and Tukey's test was used to assess corner test and rotarod test. Unpaired t-tests analyzed differences between two groups: without correction for equal SDs and with Welch's correction for unequal SDs. A significance level of *P* < 0.05 was applied throughout.

## Supplementary Material

Supplementary methods, figures and tables.
