# Supplementary material for: Neutrophil extracellular traps aggravate neuronal apoptosis and neuroinflammation via neddylation after traumatic brain injury
Source: Theranostics. 2025 Jun 20;15(15):7327–45. doi: 10.7150/thno.111512 (PMC12315821; doi:10.7150/thno.111512)
Supplement: Supplementary file 1 — Supplementary methods, figures and tables. [file thnov15p7327s1.pdf]

Part 1: Experimental flow chart and experimental design

Experimental flow chart:

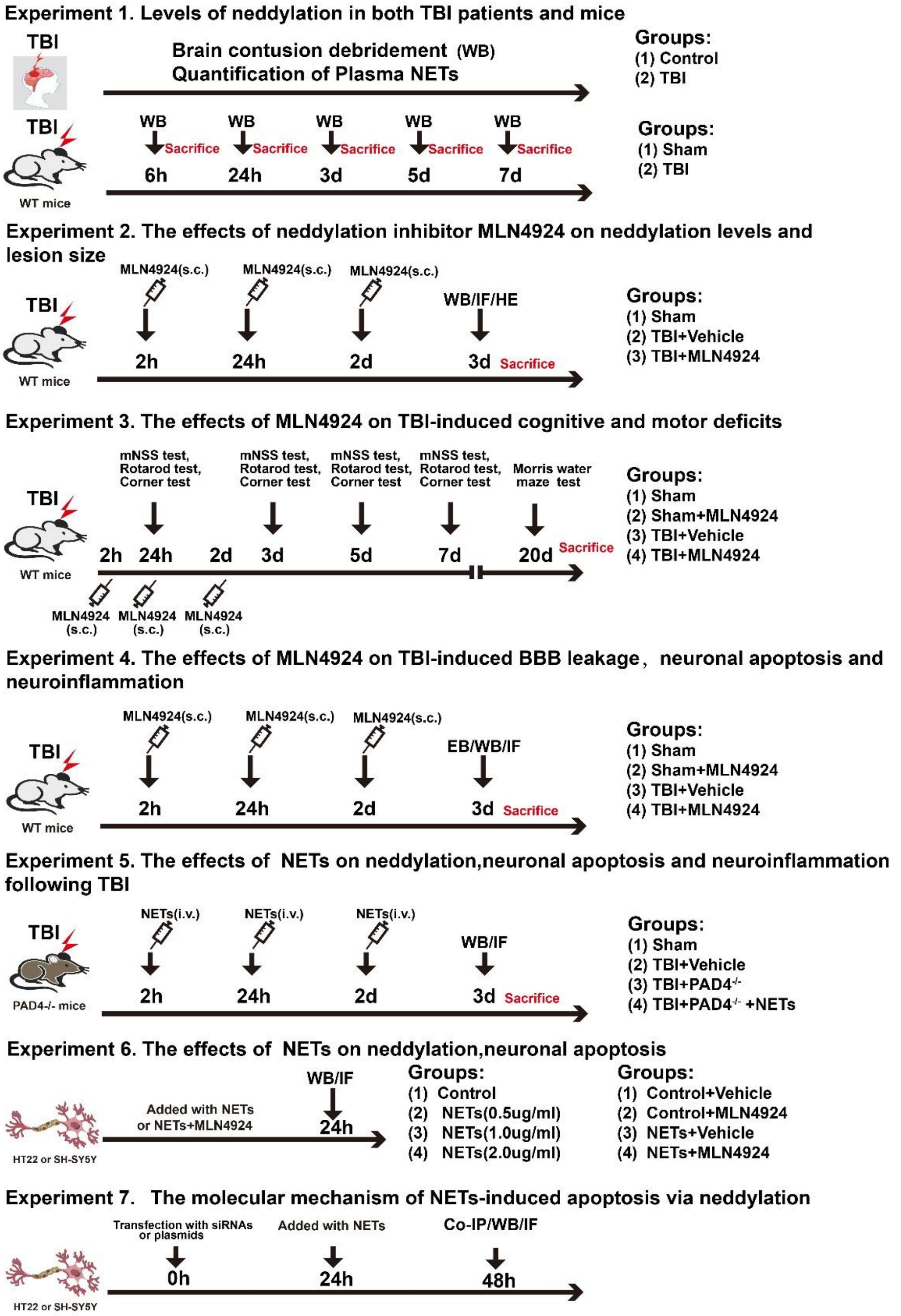

Experimental flow chart of this study. TBI: traumatic brain injury; WB: western blotting; IF: immunofluorescence; BBB: blood-brain barrier; mNSS: modified neurological severity score; s.c.: subcutaneous; PAD4: peptidyl arginine deiminase 4;

i.v. : intravenous; NETs: neutrophil extracellular traps.

Experimental design

Human samples: Due to ethical constraints and logistical limitations, the availability of clinical specimens was restricted, leading to inevitable differences in group sizes. All enrolled patients met the inclusion and exclusion criteria: Inclusion criteria: Glasgow Coma Scale (GCS) score of 3–15; diagnosis of moderate to severe TBI; age >18 years. Exclusion criteria: Presence of malignancies, major organ dysfunction or injury, pre-existing infectious or inflammatory diseases, or long-term use of immunosuppressive agents.

Animal experiments: Sample sizes varied depending on the specific experimental needs. For standard molecular and histological analyses, we used group sizes of n = 6, 8, or 10 to ensure sufficient statistical power while adhering to the 3R principles (Replacement, Reduction, and Refinement) for animal research. For behavioral experiments, a group size of n = 10 was selected to account for inter-individual variability and to enhance the robustness of behavioral outcome measures. All mice were randomly assigned to the following experiments in the present study.

Experiment 1

To investigate neddylation following TBI, we collected epileptogenic tissues from patients (n = 3) and brain tissues from TBI patients (n = 8), along with cortical tissue from CCI model mice at 0 h (Sham), 6h, 24h, 3d, 5d, and 7d post-injury (n = 6/group) for Western blotting (WB). Human circulating dsDNA and citrullinated histone H3 (H3Cit) levels were measured to quantify NETs (control = 8, TBI = 32).

Experiment 2

To determine the effects of neddylation inhibitor MLN4924 on neddylation levels and lesion size at 3d after TBI. 66 mice were randomly assigned into three groups: Sham, TBI + Vehicle, and TBI + MLN4924 (n = 18/group). 36 mice were used for WB and immunofluorescence (IF) (n = 6/group) to evaluate neddylation levels after TBI. In another set of experiments, additional 30 mice were randomly assigned into three groups (n = 10/group): Sham, TBI + Vehicle, and TBI +mln4924 and used for HE staining to evaluate lesion size after TBI.

Experiment 3

To assess the effects of MLN4924 on neurological function after TBI, 40 mice were randomly assigned into four groups: Sham, Sham + MLN4924, TBI + Vehicle and TBI + MLN4924 (n = 10/group). We conducted behavioral tests on the four groups of mice, including the modified neurological severity score (mNSS), rotarod, corner and Morris water maze (MWM) tests.

Experiment 4

To explore the effects of MLN4924 on TBl-induced BBB leakage, neuronal apoptosis and neuroinflammation.80 mice were randomly assigned into four groups: Sham, Sham + MLN4924, TBI + Vehicle and TBI + MLN4924 (n = 20/group). 32 mice were used for Evans Blue experiment (n = 8/group) after TBI. 48 mice were used for WB and IF (n = 6/group).

Experiment 5

To assess the effects of NETs on neddylation, neuronal apoptosis and neuroinflammation following TBI. 48 mice were randomly assigned into four groups: Sham, TBI + Vehicle, TBI + PAD4$^{-/-}$ and TBI + PAD4$^{-/-}$+NETs (n = 12/group). We conducted WB and IF experiments on the four groups of mice (n = 6/group).

Experiment 6

To examine the effects of NETs on neddylation and apoptosis in HT-22 and SH-SY5Y cell lines, we first treated the cells with varying concentrations of NETs (n = 3/group) and assessed neddylation levels. Subsequently, the cells were divided into four experimental groups (Control+Vehicle, Control+MLN4924, NETs+Vehicle and NET+MLN4924, n = 3/group) to investigate the impact of neddylation inhibition on NETs-induced neuronal apoptosis.

Experiment 7

To investigate the molecular mechanism of NETs-induced apoptosis via neddylation, we first transfected HT-22 or SH-SY5Y cells with siRNAs or plasmids, followed by mechanistic exploration using Co-IP, WB and IF assays (n = 3/group).

Part 2: Supplementary tables

TableS1. Modified neurological severity scores

Tests	Score
**Motor tests (Normal = 0; maximum = 6)**	
**Raising the mouse by the tail**	
Flexion of forelimb	1
Flexion of hindlimb	1
Head moving >10° to vertical axis with 30 seconds	1
**Placing the mouse on the floor**	
Inability to walk straight	1
Circling toward the paretic side	2
Falling down to the paretic side	3
**Beam balance tests (Normal = 0; maximum = 6)**	
Grasps the side of the beam	1
Hugs the beam and 1 limb falls down from the beam	2
Hugs the beam and 2 limbs fall down, or spins on the beam (> 30 seconds)	3
Attempts to balance on the beam but falls off (> 20 seconds)	4
Attempts to balance on the beam but falls off (> 10 seconds)	5
Falls off: no attempt to balance or hang on to the beam (< 10 seconds)	6
**Reflex absent and abnormal movement test (Normal = 0; maximum = 2)**	
Pinna reflex (a head shake when touching the auditory meatus)	1
Corneal reflex (an eye blink when touching the cornea with cotton)	1
Maximum points	14

A point is given for failure to complete tasks or a lack of reflex response. Scores of 10 to 14 indicate severe injury, 5 to 9 indicate moderate injury, and 1 to 4 indicate mild injury.

Table S2. Antibodies for IF assay

Primary antibodies	Cat. No.	Manufacturer
NEDD8	sc-373741	Santa Cruz Biotechnology
NEDD8	ab81264	Abcam
F4/80	ab6640	Abcam
CD31	AF3628	R&D Systems
NeuN	ab177487	Abcam
Iba-1	17198	Cell Signaling Technology (Danvers, US)
GFAP	ab7260	Abcam
CD16/32	AF1460	R&D Systems
ZO-1	61-7300	Thermo Fisher
TRIM56	ab154862	Abcam
STING	66680-1-Ig	Proteintech

Secondary antibodies	Cat. No.	Manufacturer

Alexa Fluor 594 Donkey Anti-Rabbit IgG	ab150076	Abcam
Alexa Fluor 488 Donkey Anti-Mouse IgG	ab150105	Abcam
Alexa Fluor 488 Donkey Anti-Rabbit IgG	ab150073	Abcam
Alexa Fluor 594 Donkey Anti-Mouse IgG	ab150105	Abcam
Alexa Fluor 488 Donkey Anti-Goat IgG	ab150132	Abcam

Table S3. Antibodies for Western blot

Primary antibodies	Cat. No.	Manufacturer
NEDD8	sc-373741	Santa Cruz Biotechnology
NAE1	ab187142	Abcam
UBA3	ab124728	Abcam
UBC12	ab109507	Abcam
β-actin	3700	Cell Signaling Technology
iNOS	ab178945	Abcam
Arg-1	93668	Cell Signaling Technology
Bax	2772	Cell Signaling Technology
Cleaved caspase-3	9661	Cell Signaling Technology
Cleaved caspase-7	9491	Cell Signaling Technology
ZO-1	61-7300	Thermo Fisher
VE-cadherin	Ab33168	Abcam
Occludin	71-1500	Thermo Fisher
ICAM-1	ab222736	Abcam
p-p65	3033	Cell Signaling Technology
P65	8242	Cell Signaling Technology

Secondary antibodies	Cat. No.	Manufacturer
HRP-linked anti-rabbit IgG	7074	Cell Signaling Technology
HRP-linked anti- mouse IgG	7076	Cell Signaling Technology

Table S4. Antibodies for co-IP and subsequent immunoblot assays

Antibodies	Cat. No.	Manufacturer
TRIM56	ab154862	Abcam
TRIM56	MA5-27076	Thermo Fisher
NEDD8	sc-373741	Santa Cruz Biotechnology
K63-linked ubiquitin	ab179434	Abcam
STING	ab239074	Abcam
STING	66680-1-Ig	Proteintech
Flag	14793	Cell Signaling Technology
Flag	8146	Cell Signaling Technology
HA	3724	Cell Signaling Technology
HA	2367	Cell Signaling Technology
Myc	2276	Cell Signaling Technology
Myc	2272	Cell Signaling Technology

Table S5. Demographic and clinical characteristics of patients.

TBI patient	Accident type	Lesion	Gender	Age	GCS scores
1	Fall	Right temporal lobe	Male	47	8
2	Traffic accident	Right temporal lobe	Female	56	6
3	Traffic accident	Left temporal lobe	Female	59	9
4	Traffic accident	Occipital lobe	Female	37	8
5	Traffic accident	Left temporal lobe	Female	45	7
6	Fall	Occipital lobe	Male	56	7
7	Fall	Left temporal lobe	Male	33	9
8	Traffic accident	Left temporal lobe	Male	48	6
**Epilepsy patient**	**Epilepsy type**	**Lesion**	**Gender**	**Age**	**duration of illness(years)**
1	drug-resistant epilepsy	Left temporal lobe	Female	50	2.5
2	drug-resistant epilepsy	Left temporal lobe	Male	55	3
3	drug-resistant epilepsy	Right temporal lobe	Male	47	3

Part 3: Supplementary figures

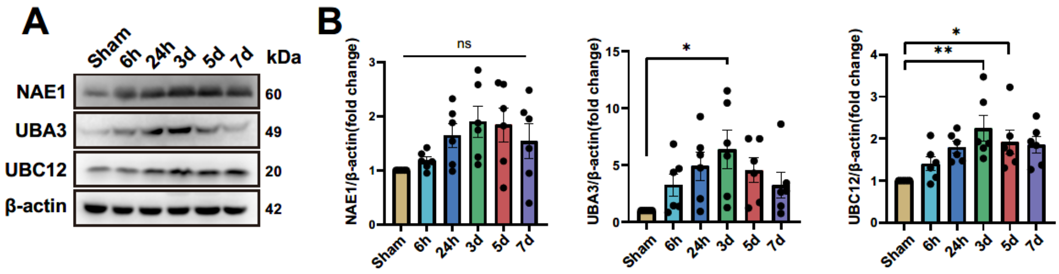

Figure S1. Elevated expression of neddylation-related enzymes. (A-B) Quantitative immunoblot analysis of NAE1, UBA3, and UBC12 expression post-TBI at various time points. n = 6 per group. Statistical comparisons among multiple groups were performed using one-way ANOVA test. **P* < 0.05, ***P* < 0.01. Data are presented as mean values ± SEM.

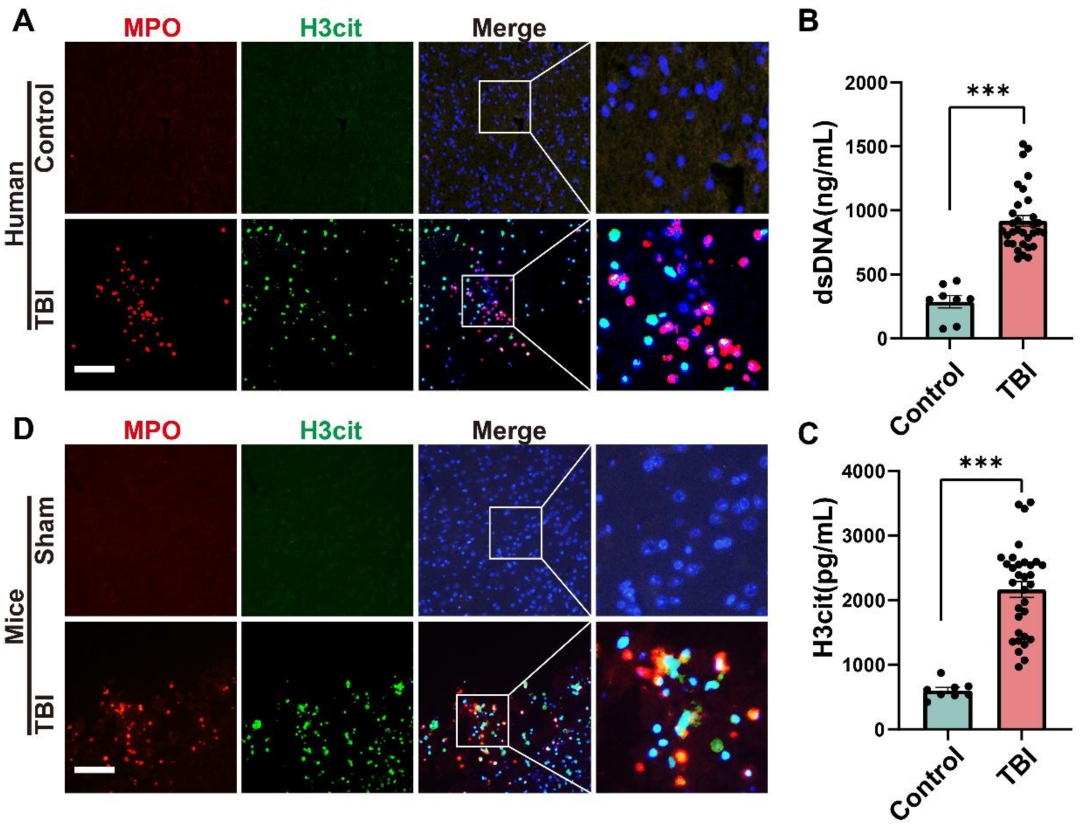

Figure S2. Formation of NETs Following TBI. (A) Representative images illustrating the colocalization of neutrophils (MPO, red) and citrullinated histone H3 (H3cit, green) at the lesion site in human brain tissues following TBI. Scale bar = 50 μm. (B) Plasma levels of double-stranded DNA (dsDNA) in controls (n = 8) and patients with TBI (n = 32). Statistical analysis was performed using an unpaired Student's t-test. (C) Plasma levels of H3cit in controls (n = 8) and patients with TBI (n = 32). Statistical analysis was performed using an unpaired Student's t-test. (D) Representative images showing colocalization of neutrophils (MPO, red) and citrullinated histone H3 (H3cit, green) at the lesion site in mouse brain tissue 3 days after TBI. Scale bar = 50 μm.

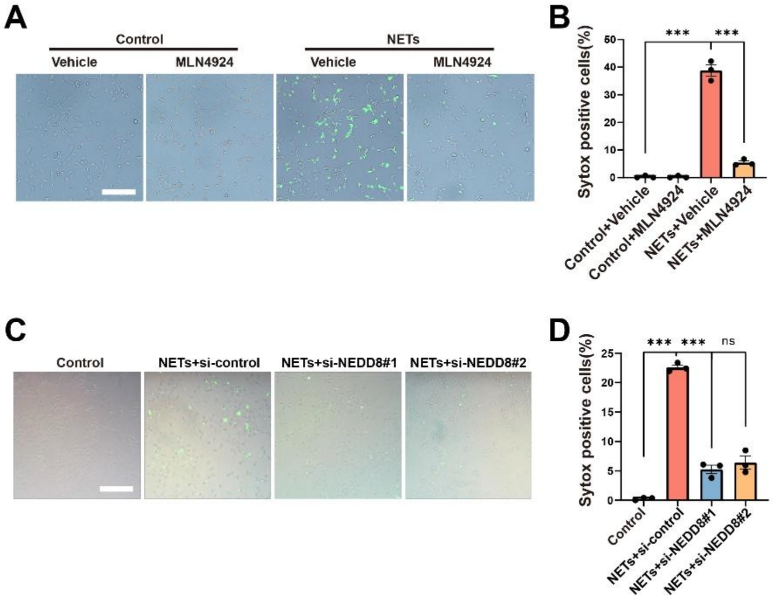

Figure S3. Sytox staining of HT22 cells. (A) Representative images of SYTOX (green) staining in HT22 cells treated with NETs or NETs combined with MLN4924. Scale bar = 100 µm. (B) Quantitative analysis of the proportion of SYTOX-positive cells across different groups. n = 3 per group. (C) Images of SYTOX (green) staining in NETs-exposed HT22 cells transfected with si-NEDD8 or si-control. Scale bar = 100 µm. (D) Bar graph represents the percentage of SYTOX-positive cells across groups. n = 3 per group. ***$P < 0.001$. Statistical comparisons among multiple groups were performed using one-way ANOVA test. Data are presented as mean values ± SEM.

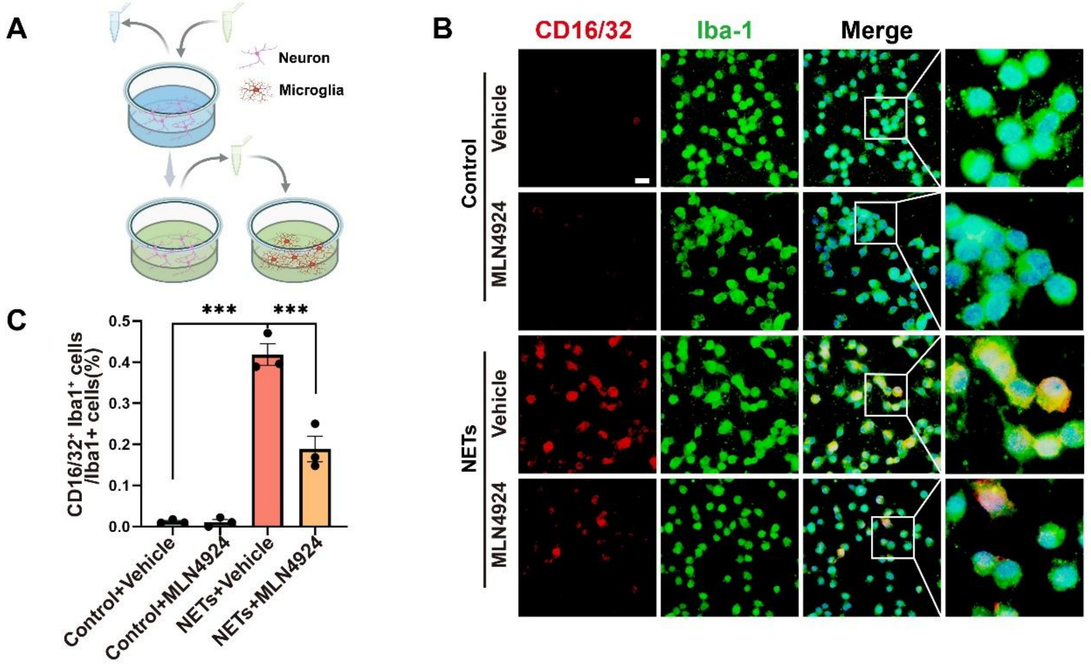

Figure S4. Neddylation in neurons modulates the microglial neuroinflammatory response. (A) Experimental workflow created with BioRender. HT22 cells were treated with NETs, with or without MLN4924, for 24 hours. The medium was then replaced, and cells were cultured for another 24 hours. The conditioned medium was

subsequently applied to BV2 cells for overnight incubation. (B) Immunofluorescence analysis of CD16/32(red) expression in BV2 cells (Iba-1, green) after 24-hour exposure to neuronal-conditioned media under different conditions. Scale bar = 20 μm. (C) Quantitative analysis of CD16/32$^+$/ Iba1$^+$ microglia co-cultured with HT22 cells under different conditions. n = 3 per group. Statistical comparisons among multiple groups were performed using one-way ANOVA test. ***$P < 0.001$, Data are presented as mean values ± SEM.

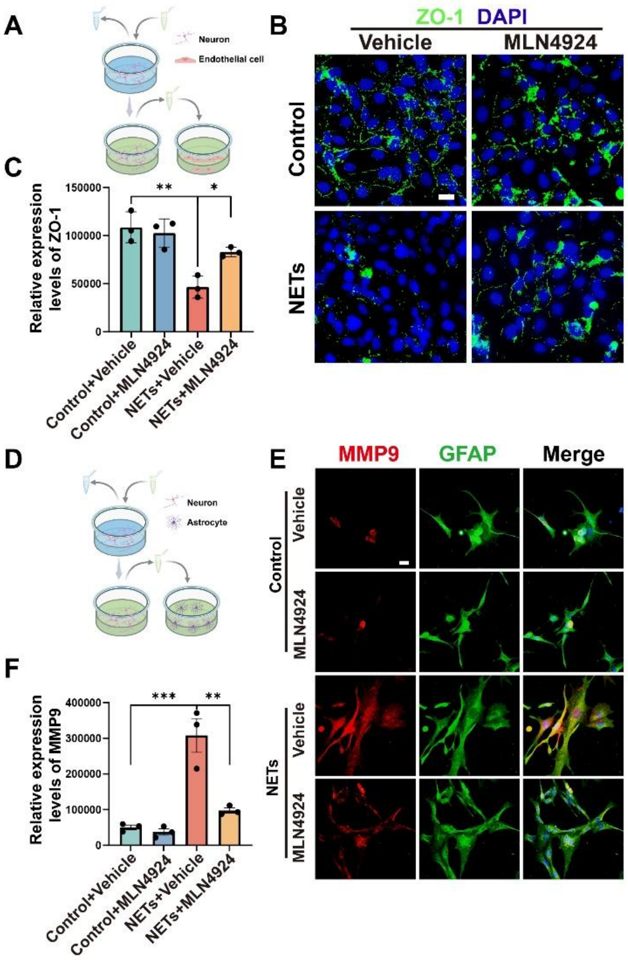

Figure S5. Neuronal neddylation mediated by NETs alters endothelial tight junction integrity and astrocytic MMP9 expression. (A) Experimental workflow created with BioRender. SH-SY5Y cells were treated with NETs, with or without MLN4924, for 24 hours. The medium was then replaced, and cells were cultured for another 24 hours. The conditioned medium was subsequently applied to human brain microvascular endothelial cells (HBMECs) for overnight incubation. (B) Representative immunofluorescence images showing ZO-1 (green) expression in HBMECs after 24-hour exposure to neuron-conditioned media under different experimental conditions. Scale bar = 20 μm. (C) Quantitative analysis of relative ZO-1 expression levels under various conditions. (D) Experimental workflow created with BioRender.HT22 cells were treated with NETs, with or without MLN4924, for 24 hours. The medium was then replaced, and cells were cultured for another 24 hours. The conditioned medium was subsequently applied to C8-D1A cells for overnight incubation. (E) Representative immunofluorescence images showing MMP9 (red) expression in C8-D1A cells after 24-hour exposure to neuron-conditioned media under

different experimental conditions. Scale bar = 20 μm. (F) Quantitative analysis of relative MMP9 expression levels under various conditions. n = 3 per group. Statistical comparisons among multiple groups were performed using one-way ANOVA test. Data are presented as mean ± SEM. *$P < 0.05$, **$P < 0.01$, ***$P < 0.001$.

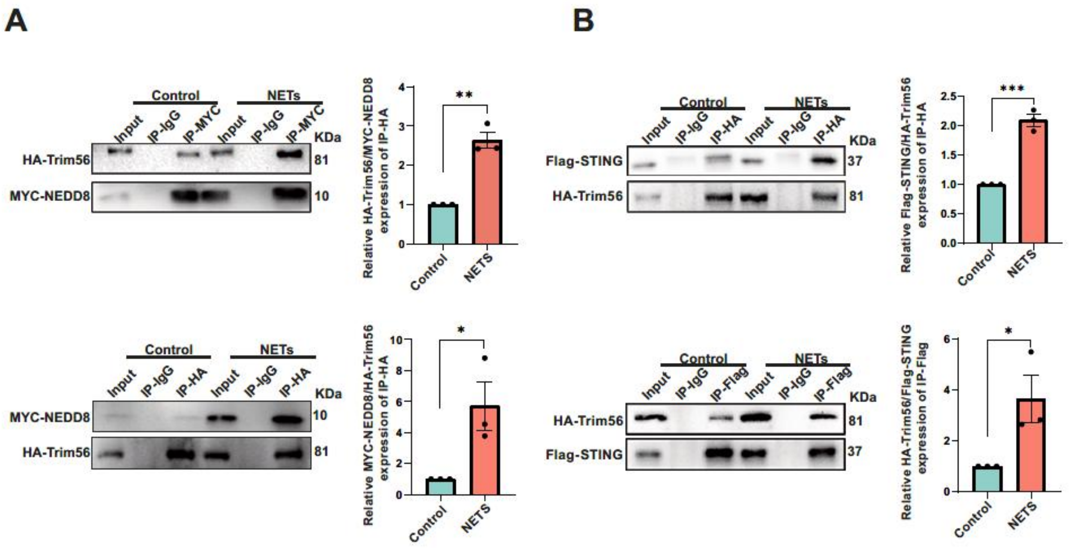

Figure S6. Mouse-derived NETs enhance NEDD8-TRIM56 and TRIM56-STING interactions in HT22 cells. (A) Co-IP analysis and quantification revealing the effect of NETs on the NEDD8-TRIM56 interaction. (B) Co-IP analysis and quantification showing the effect of NETs on the TRIM56-STING interaction. n = 3 per group. Statistical analysis was performed using an unpaired Student's t-test. *$P < 0.05$, **$P < 0.01$, ***$P < 0.001$, Data are presented as mean values ± SEM.

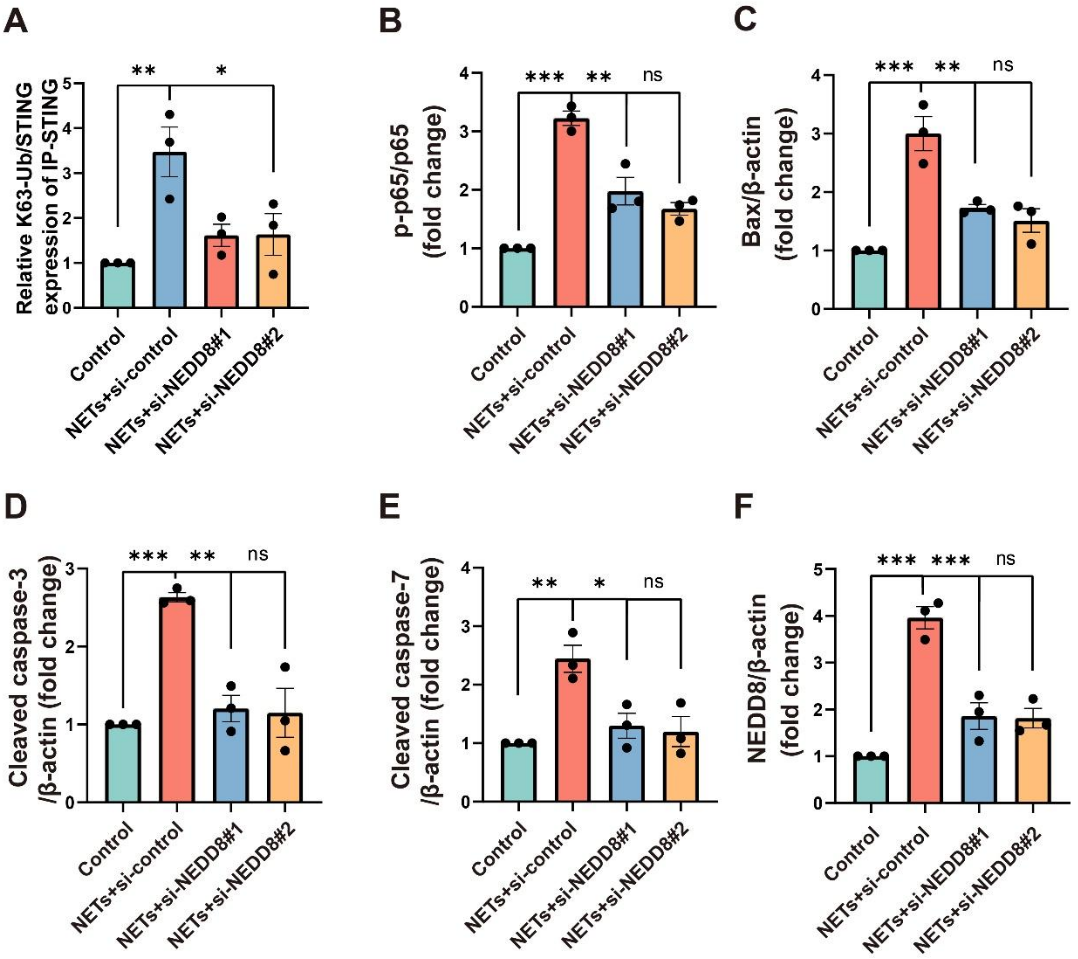

Figure S7. The role of NEDD8 in mediating STING K63-linked ubiquitination and neuronal apoptosis in SH-SY5Y cells induced by human-derived NETs. (A) Quantitative analysis of K63-Ub. (B-F) Quantitative analysis of p-p65 (B), Bax (C), Cleaved caspase-7 (D), Cleaved caspase-3 (E) and NEDD8 (F). n = 3 per group. Statistical comparisons among multiple groups were performed using one-way ANOVA test. **P* < 0.05, ***P* < 0.01, ****P* < 0.001, Data are presented as mean values ± SEM.

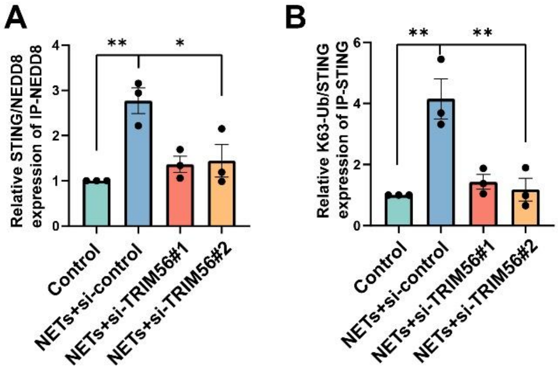

Figure S8. The role of TRIM56 in mediating NEDD8-STING binding and STING K63-linked ubiquitination in SH-SY5Y cells induced by human-derived NETs. (A) Quantitative analysis of STING. (B) Quantitative analysis of K63-Ub. n = 3 per group. **P* < 0.05, ***P* < 0.01. Statistical comparisons among multiple groups were performed using one-way ANOVA test. Data are presented as mean values ± SEM.

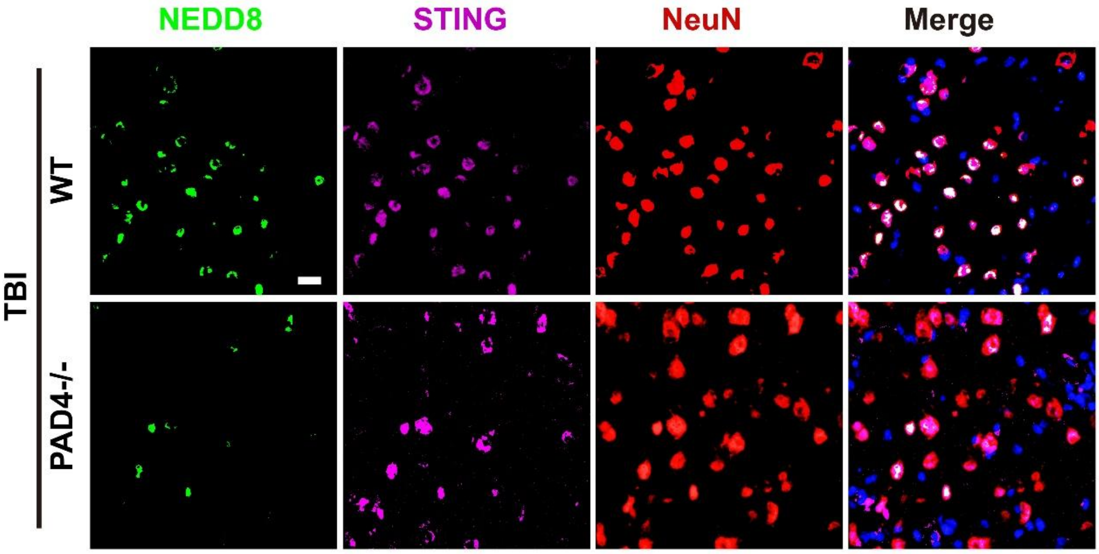

Figure S9. Representative IF images of NEDD8, STING, and NeuN co-staining in TBI and TBI+PAD4(-/-) mice. Scale bar = 20 μm.
